# Cost-effectiveness analysis of COVID-19 variants effects in an age-structured model

**DOI:** 10.1038/s41598-023-41876-x

**Published:** 2023-09-22

**Authors:** Giphil Cho, Young Jin Kim, Sang-hyup Seo, Geunsoo Jang, Hyojung Lee

**Affiliations:** 1 Department of Artificial Intelligence and Software, Kangwon National University, Chuncheon, Gangwon 25913 Republic of Korea; 2 Division of Data Analysis, Center for Global R&D Data Analysis, Korea Institute of Science and Technology Information (KISTI), Seoul, 02456 Republic of Korea; 3 National Institute for Mathematical Sciences, Daejeon, 34047 Republic of Korea; 4 Nonlinear Dynamics and Mathematical Application Center, Kyungpook National University, Daegu, 41566 Republic of Korea; 5 Department of Statistics, Kyungpook National University, Daegu, 41566 Republic of Korea

**Keywords:** Viral infection, Computational models

## Abstract

This study analyzes the impact of COVID-19 variants on cost-effectiveness across age groups, considering vaccination efforts and nonpharmaceutical interventions in Republic of Korea. We aim to assess the costs needed to reduce COVID-19 cases and deaths using age-structured model. The proposed age-structured model analyzes COVID-19 transmission dynamics, evaluates vaccination effectiveness, and assesses the impact of the Delta and Omicron variants. The model is fitted using data from the Republic of Korea between February 2021 and November 2022. The cost-effectiveness of interventions, medical costs, and the cost of death for different age groups are evaluated through analysis. The impact of different variants on cases and deaths is also analyzed, with the Omicron variant increasing transmission rates and decreasing case-fatality rates compared to the Delta variant. The cost of interventions and deaths is higher for older age groups during both outbreaks, with the Omicron outbreak resulting in a higher overall cost due to increased medical costs and interventions. This analysis shows that the daily cost per person for both the Delta and Omicron variants falls within a similar range of approximately $10–$35. This highlights the importance of conducting cost-effect analyses when evaluating the impact of COVID-19 variants.

## Introduction

Originating in Wuhan, China in December 2019, coronavirus disease 2019 (COVID-19) swiftly spread worldwide, leading to confirmed cases in nearly every country and emerging as a significant global public health crisis^1, 2^. COVID-19 is caused when someone is infected by severe acute respiratory syndrome coronavirus 2 (SARS-CoV-2). COVID-19 continues to spread vigorously in several countries; as of December 31, 2022, there were 729,470,516 confirmed COVID-19 cases and 6,718,923 deaths reported worldwide^3^. The control strategies for COVID-19 include nonpharmaceutical interventions (NPIs) and vaccination programs. Different NPIs have been employed to reduce COVID-19 cases, including social distancing, wearing of masks, temporary shutdowns in schools, reductions in social activities, and employment-related restrictions^4, 5^. NPIs are crucial in controlling COVID-19 infections, especially when effective vaccines are unavailable. Mass vaccination against COVID-19 began in Europe at the end of 2021. Most governments in Europe continued to impose limitations on social activities in tandem with their vaccination programs^6, 7^.

Previous studies have utilized age-structured deterministic transmission models to investigate the effects of NPIs on the COVID-19 pandemic^8^. Vital measures must account for age-dependent transmissions because NPIs (e.g., school and workplace closures) influence contact patterns between age groups, and vaccination is related to varied age groups. Prem et al.^8^ employed an age-structured susceptible-exposed-infected-removed (SEIR) model to investigate several physical distancing measures under varying control scenarios in Wuhan, China. Jaouimaa et al.^9^ proposed an age-structured deterministic SEIR model to describe the community spread of COVID-19, incorporating the impact of age-specific social interactions in an Irish context. Viana et al.^10^ used an age-structured transmission model to evaluate the impact of relaxation scenarios. They projected hospital admissions, time-dependent effective reproduction numbers, and the timing of achieving control over COVID-19 in Portugal. As observed, the continuation of socioeconomic activities can create new waves of the pandemic.

Additionally, fractional differential equations for dynamical systems, machine learning methods, and statistical methods have been used to predict the transmission dynamics. Fractional calculus approaches precisely describe COVID-19 transmission^11–14^. Statistical approaches such as Auto-Regressive Integrated Moving Average Model (ARIMA) are employed to forecast COVID-19 cases, deaths, and recovered cases^15, 16^. Machine learning methods help detect changes in transmission trends^17–19^.

The first COVID-19 case in the Republic of Korea was diagnosed on 20 January 2020^20^. Subsequently, the country was afflicted by the COVID-19 pandemic, experiencing more than five waves of infections between February 2020 and December 2022. When the pandemic was first announced, the Korean government implemented NPIs, limiting or prohibiting numerous social activities and gatherings, and frequently closing schools. These policies were relaxed or strengthened in accordance with the spread of infections. The second and third waves of COVID-19 occurred in August and November 2020, respectively, evidently indicating that NPIs could not adequately control the pandemic. The Korean government launched its vaccination program on February 26, 2021. Nevertheless, the fourth wave began in July 2021 and continued until March 2022 because of the high transmissibility of a new variant of the virus.

The emergence and dissemination of COVID-19 variants such as Delta and Omicron despite all efforts to prevent and control the disease resulted in a continuous increase in the number of infected individuals^21, 22^. Pandemic control efforts were pivotally driven by the emergence of concerning variants, particularly the lineages of Alpha (B.1.351), Delta (B.1.617.2), and Omicron (B.1.1.529). The Delta and Omicron variants rapidly became dominant strains, accounting for over 90% of total COVID-19 cases in the Republic of Korea in August 2021 and January 2022, respectively. The Delta variant was substantially more transmissible and caused higher mortality than the alpha variants (pre-Delta)^23^. Consequently, several countries currently confront the potential risk of additional COVID-19 outbreak waves.

The assessment of the costs associated with vaccination efforts and NPIs is crucial for the effective control of COVID-19. Age-group-based control interventions against COVID-19 have been found to demonstrate limited health and economic impact^24–26^. However, only a few investigations have probed cost-effectiveness using mathematical models. Most such studies have focused primarily on economic influence, neglecting the heterogeneity and impact of NPIs^25, 27^. Effective control interventions are necessary, not only to reduce increasing COVID-19 infections but also to mitigate rising fatalities (deaths) because both factors pose significant risks. The fatality rate is higher among people over the age of 65 years, making it crucial to analyze the costs of reducing infections and deaths across discrete age groups.

Our study aimed to analyze how COVID-19 variants affected cost-effectiveness in discrete and vaccine-eligible age groups. We proposed an age-structured compartmental model to describe COVID–19 transmission, incorporating the impact of age-specific social interactions. Our model focused on COVID-19 transmission in the Republic of Korea, but it can be adapted for other countries by using relevant parameters.

## Results

### Parameter estimation

**Figure 1:**
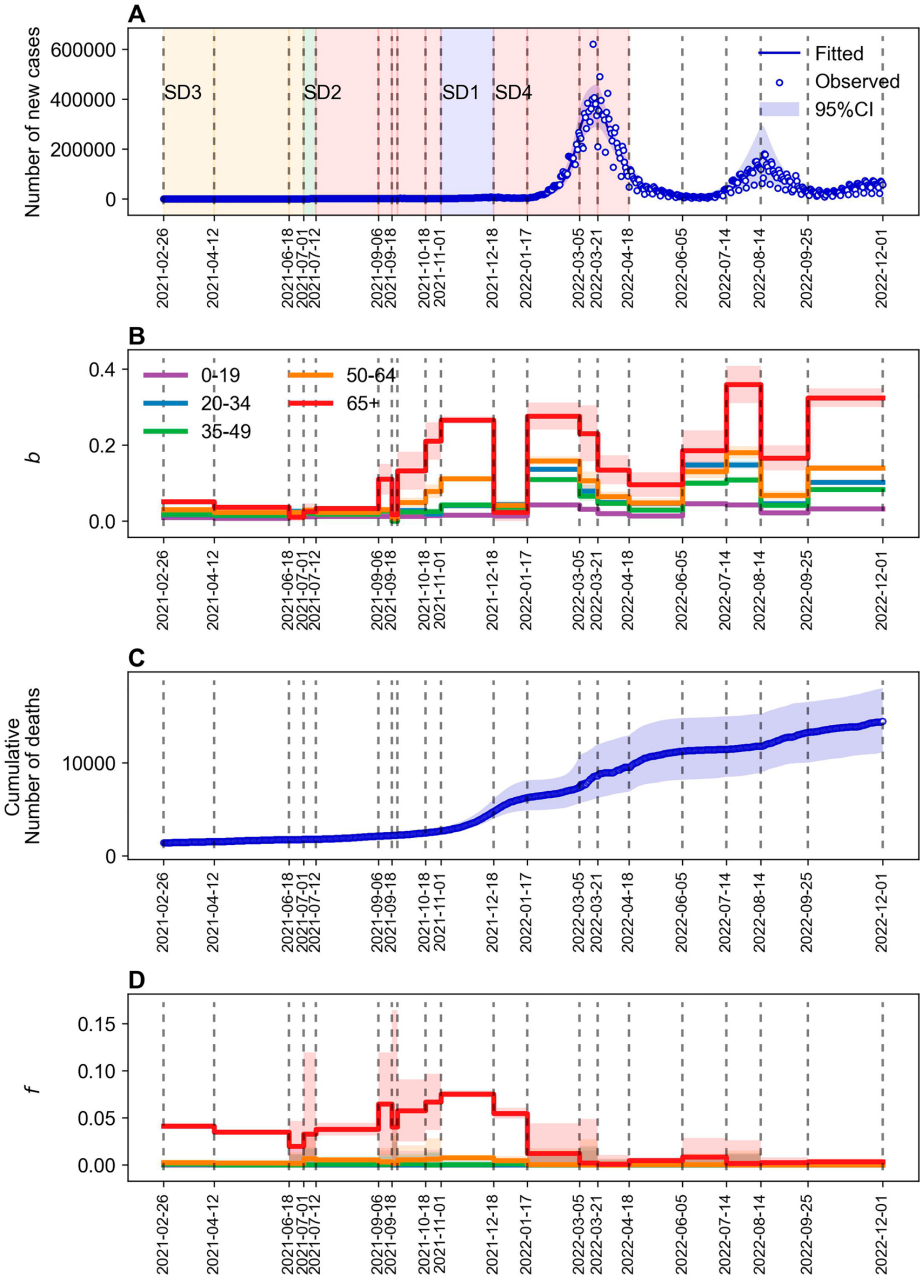
Model fit to COVID-19 reported cases and deaths. The blue-solid lines represent the estimated values obtained using the age-structured model. The blue-shaded region represents the 95%-confidence intervals based on 1,000 parameter samples. The vertical gray-dashed lines denote the time periods ($P_{i}$, $i = 1, \ldots ,19$). From the data, the blue dots in (**A**) and (**C**) denote the daily reported cases and cumulative deaths, respectively. Fitted values of ${\varvec{b}}$ and ${\varvec{f}}$ for each age group are presented in (**B**) and (**D**), respectively.

Figure 1 displays the estimated values of the transmission rates ${\varvec{b}}$ and case-fatality rates ${\varvec{f}}$ based on age groups. The observed and estimated cases were classified according to age groups. As observed, the majority of the fitted values for the cases and the cumulative number of deaths were plotted within 95% confidence intervals. Figure 1A presents a comparison of the values estimated from the model based on the transmission rates ($b$) with the observed COVID-19 cases. Figure 1B depicts the time-varying transmission rates segmented according to age groups. Supplementary Fig. 1 presents a comparison of the estimated values by age groups against observed cases and fatalities. We distinguished transmission rates (${b}_{i}$) and case-fatality rates (${f}_{i}$) based on age group *i*. Supplementary Table 1 summarizes all the estimated values for the various age groups. Specifically, the 65+ age group exhibited high transmission and case-fatality rates. Transmission rates were estimated as 0.0000–0.0461 for the 0–19 age group and 0.0102–0.3595 for the 65+ age group. The transmission rate was comparatively higher (0.0956–0.3595) in individuals classified to the 65+ age group than among individuals in other age groups (0.0134–0.1803) when the Omicron variant was dominant (approximately 90%) in periods ${P}_{12}-{P}_{19}$.

The cumulative number of deaths ascertained from the data was compared to the values estimated by the model using case-fatality rates ($f$) recorded in Fig. 1C (please also refer to Fig. 1D). The estimated case-fatality rates were high in all periods only in the 65+ age group; conversely, case-fatality rates were estimated as almost zero for the 0–19 age group. The case-fatality rates for the 50–64 and 65+ age groups were estimated as 0.0018–0.0078 and 0.0378–0.0752, respectively, when the Delta variant cases exceeded 90% in periods ${P}_{5}-{P}_{10}$. This outcome indicated that case-fatality rates increased from the previously estimated values of 0.0018–0.0068 and 0.0197–0.0412 in ${P}_{4}$ because of the impact of the Delta variant. Therefore, the transmission of Delta and Omicron variants is crucial for determining the transmission rates and case-fatality rates of COVID-19.

### Impact of variants on COVID-19 cases and deaths

**Figure 2:**
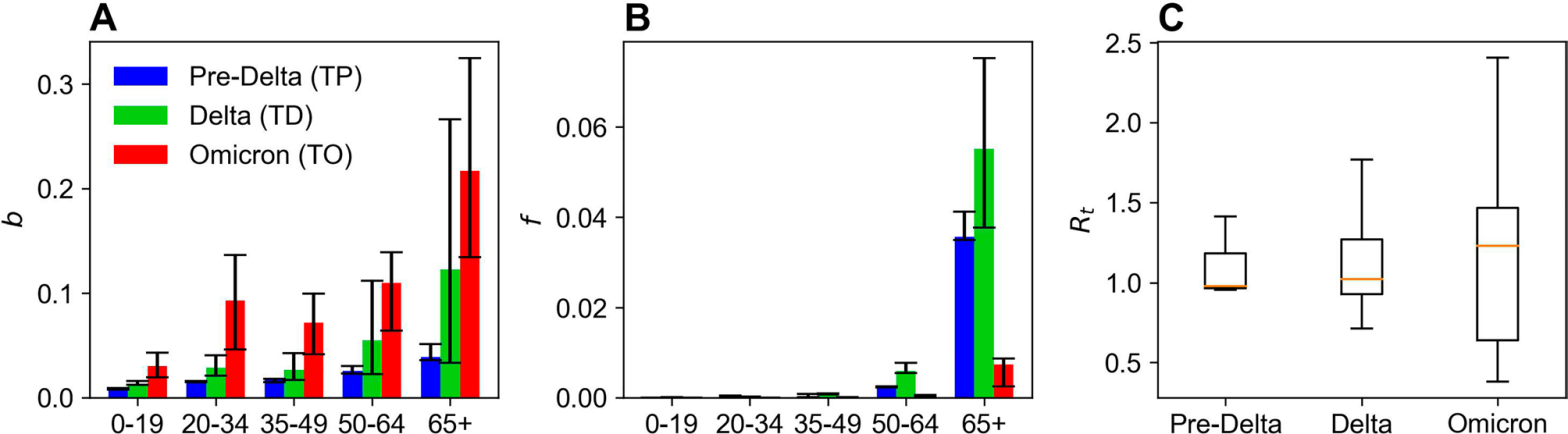
Estimated parameters according to the three phases by age groups. Error bars depict the range between 25 and 75th percentiles. (**A**) Average transmission rates by age groups during each phase. (**B**) Average case-fatality rates by age groups during each phase. (**C**) Effective reproduction number during each phase. Orange horizontal lines represent the average values.

We estimated the transmission and case-fatality rates for the 19 established time intervals using the number of COVID-19 cases and deaths by age group. We subsequently compared the age-specific transmission rates ($\mathbf{b}$), case-fatality rates (***f***)*,* and the effective reproduction number (${R}_{\mathrm{t}}$) values during TP, TD, and TO, as presented in Fig. 2 and Table 1. Overall, the Omicron variants increased transmission rates and decreased case-fatality rates. Figure 2A shows the estimated transmission rates by comparing TP, TD, and TO. The transmission rate of TO (Omicron variant) increased approximately two-fold over the TD phase when the Delta variant was dominant. Specifically, COVID-19 transmission increased by around 3.25 times in the 20–34 age group. The case-fatality rate in the Omicron variant decreased by approximately 0.1 times for age groups 35 and older in comparison to the Delta variant, as elucidated in Fig. 2B.

**Table 1:** Variant-specific average transmission rates, case-fatality rates, total costs, and effective reproduction numbers from February 26, 2021, to November 30, 2022, classified by age groups according to TP, TD, and TO for each parameter.

Parameters	Period	0–19	20–34	35–49	50–64	65+
$\overline{{\varvec{b}} }$	TP	0.0085 (0.65)	0.0162 (0.57)	0.0168 (0.63)	0.0256 (0.46)	0.0389 (0.32)
	TD	0.0130 (–)	0.0286 (–)	0.0265 (–)	0.0551 (–)	0.1229 (–)
	TO	0.0303 (2.33)	0.0930 (3.25)	0.0717 (2.71)	0.1099 (1.99)	0.2170 (1.77)
$\overline{{\varvec{f}} }$	TP	0.0000 (1.00)	0.0002 (1.00)	0.0003 (0.33)	0.0023 (0.38)	0.0357 (0.65)
	TD	0.0000 (–)	0.0002 (–)	0.0009 (–)	0.0060 (–)	0.0551 (–)
	TO	0.0000 (1.00)	0.0000 (0.00)	0.0001 (0.11)	0.0006 (0.1)	0.0074 (0.13)
Total cost	TP	0.1845 (0.13)	0.3080 (0.2)	0.2838 (0.2)	0.2449 (0.19)	0.2464 (0.14)
	TD	1.4686 (–)	1.5652 (–)	1.4354 (–)	1.2658 (–)	1.7096 (–)
	TO	54.4068 (37.05)	50.4786 (32.25)	45.9175 (31.99)	26.8918 (21.24)	16.7845 (9.82)
${R}_{t}$	TP	1.0908 (1.01)				
	TD	1.0802 (–)				
	TO	1.1292 (1.05)				

(∙) indicates the multiplier compared to the values in the Delta phase (TD).

The case-fatality rate was significantly high for the Delta variant for 65+ individuals. Age group comparisons revealed that 65+ individuals evinced a wide range of predicted values for both transmission and case-fatality rates. Comparisons of the effective reproduction number (${R}_{\mathrm{t}}$) across TP, TD, and TO disclosed a similar average ${R}_{\mathrm{t}}$ at around 1; however, the distribution was much wider during TO, when the Omicron variant was dominant.

### Evaluating variant-specific costs across age groups

**Figure 3:**
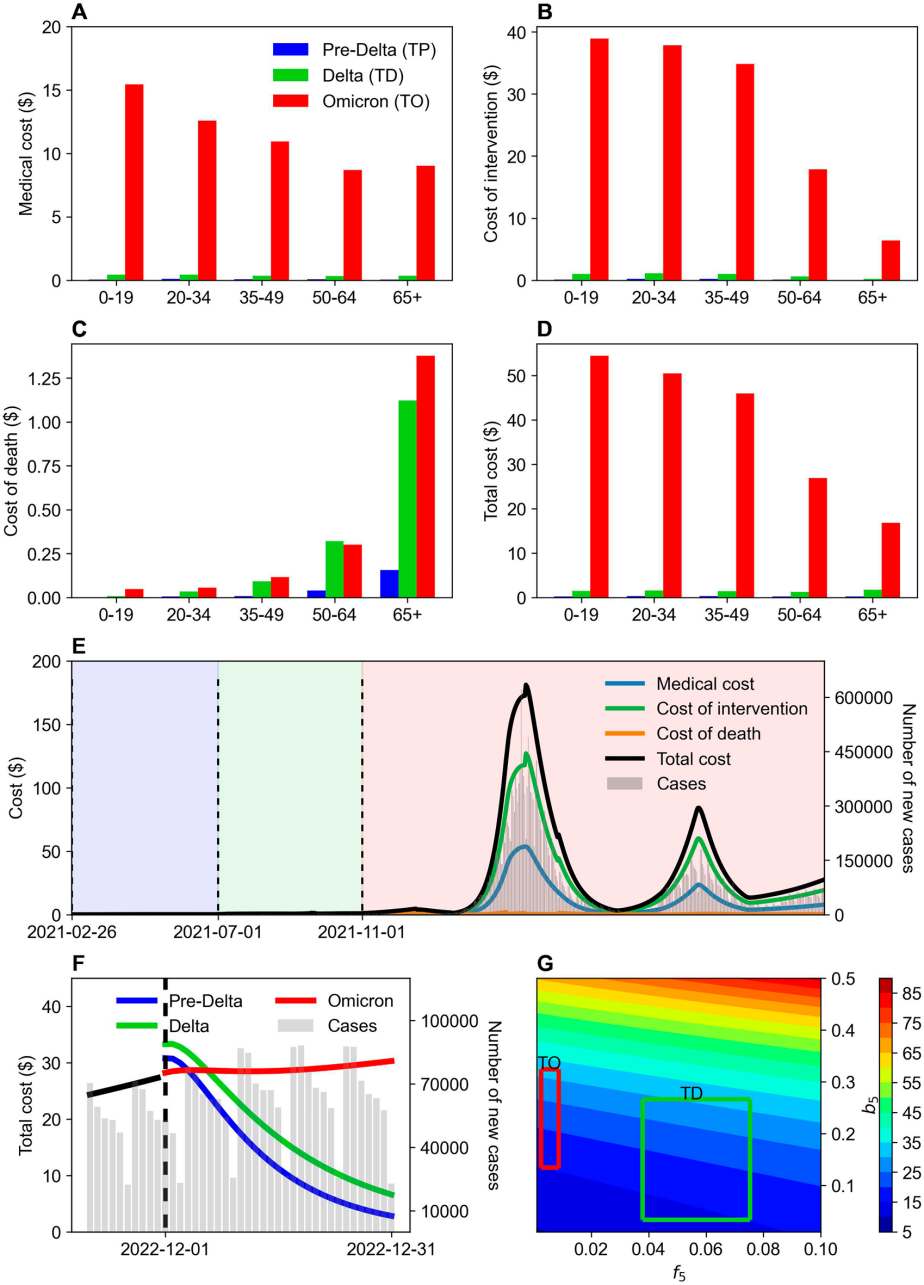
Evaluation of the cost-effectiveness across COVID-19 variants and age groups for TP, TD, and TO. Unit cost (dollars) is the cost per capita per day. (**A**)–(**D**) Medical cost, cost of intervention, cost of death, and total cost is compared by age groups and phases. (**E**) Shaded region represents the TP (blue), TD (green), and TO (red). Types of cost and total cost over time are shown from February 26, 2021 to November 30, 2022 (left y-axis). The different number of COVID-19 cases are shown in gray bars (right y-axis). Vertical dashed lines represent the 19-time intervals (${P}_{1}-{P}_{19}$). (**F**) Estimated cost for a new variant. Vertical line indicates the start time point of forecasting period. Black line is the total cost of the fitted period. Colored lines show the predicted total cost when a new variant emerged for Pre-Delta (TP), Delta (TD), and Omicron (TO). The different number of COVID-19 cases are shown in gray bars (right y-axis). (**G**) Sensitivity analysis is conducted. Total cost during a month from December 2022 is computed with varying transmission and case-fatality rates based on 65+ age group.

Figure 3 illustrates the variant-specific cost computed for the three phases TP, TD, and TO, as well as demonstrates that medical costs accounted for a significant percentage of the total costs during TO. Figure 3B evinces that the cost of intervention for the under-49 age group was much higher than the other age groups, approximately $40 per capita per day. Figure 3C indicates that the cost of death was steep during both TD and TO, particularly for the 65+ age group. Figure 3D,E show that the total cost increased significantly during TO because of the upsurging COVID-19 cases and the consequently escalating costs of medical treatments and other interventions. Table 1 elucidates that compared to TD, the cost increased more than 30 times for the under-49 age group during TO. Specifically, the cost increased 9.8 times for the 65+ age group, a lower multiplier compared to the younger age group. However, the result for the 65+ age group may be attributed to its already high baseline values during TD.

Figure 3F displays the prediction of future costs in the event of a new COVID-19 variant with characteristics similar to the pre-Delta, Delta, and Omicron strains. We estimated the number of COVID-19 cases and deaths from December 1 to 31, 2022 using average transmission rates ($$\overline{{\varvec{b}} }$$) and average case-fatality rates ($$\overline{{\varvec{f}} }$$) according to TP, TD, and TO, as summarized in Table 1. The fatality rate (***f***) was predicted to be higher than Omicron and to result in more deaths and increased costs when ***b*** and ***f*** exhibited pre-Delta and Delta values. However, the lower transmission rate (***b***) was estimated to cause fewer cases to occur, reducing the per capita daily average cost from approximately $30–$35 on December 1, 2022, to $5–$10 on December 31, 2022. However, the fatality rate was predicted to be low when ***b*** and ***f*** evinced values similar to the Omicron dominant phase. However, the high transmission rate was estimated to increase the per capita daily average cost from $28 on December 1, 2022, to $30 after a month.

Figure 3G presents a comparison of the daily cost per person when the transmission rate ($${b}_{5}$$) in the 65+ age group rises from 0.01 to 0.5, and the case-fatality rate ranges between 0.001 and 0.1. The daily cost per person was more sensitive to transmission rate changes than the fatality rate. When $${b}_{5}$$ increased by 0.01, the cost rose by approximately 9–11%; when $${f}_{5}$$ increased by 0.01, the cost amplified by approximately 3–6%. Figure 2 demonstrates that the transmission rates between the 25th and 75th percentiles (i.e., Q1–Q3) for Delta and Omicron were 0.0333–0.2661 and 0.1347–0.3245, respectively, indicating a significantly higher transmission rate for Omicron. The Q1–Q3 fatality rates for Delta and Omicron were 0.0378–0.0752 and 0.0025–0.0086, respectively, revealing a much lower fatality rate for Omicron. Given the transmission and fatality rate ranges for Delta and Omicron, the daily cost per person for both variants ranged approximately similarly between $10 $$\mathrm{and\; \$}$$35. However, Omicron imposed higher costs despite the equivalent cost range because of the surge in infections. Conversely, Delta generated increased costs because of the higher fatality rate and the consequent greater number of deaths. This outcome highlights the importance of cost-effect analyses for both infections and fatalities. This study conducted such an analysis and computed the total cost over a month from December 1 to 31, 2022 by changing the transmission and case-fatality rates based on the 65+ age group.

## Discussion

This study presented an age-structured model that considered both vaccination efforts and NPIs to evaluate the impact of COVID-19 variants such as Delta and Omicron in the Republic of Korea through cost-effectiveness across age groups. We estimated the transmission and case-fatality rates for different age groups during the COVID-19 pandemic. We examined the impact of the Delta and Omicron variants on transmission rates, case-fatality ratios, and effective reproduction numbers across age groups. We also conducted cost-effectiveness analyses for variant-specific expenditure due to the pre-Delta, Delta, and Omicron periods, providing valuable insights into their economic impact. Future costs were predicted based on the characteristics of the pre-Delta, Delta, and Omicron variants. These implications can help policymakers prepare for potential future scenarios.

Despite its contributions, we must acknowledge several limitations of our study. First, we did not consider waning vaccine immunity. Sonabend et al.^4^ employed a model that envisaged a range of optimistic-to-pessimistic vaccine effectiveness and naturally waning immunity to assess the effects of lockdown restrictions and the relaxation of COVID-19 measures balanced with the rollout of vaccination in England, UK. The present study was focused on determining the impact of variants on cost-effectiveness and not on the effects of vaccination. Therefore, waning vaccine immunity may be deemed a less influential factor. Second, we assumed a constant value for vaccine effectiveness, in alignment with a previous study^28^. However, vaccine effectiveness varies in the real world depending on the vaccine type; for instance, the efficiencies of the Pfizer-BioNTech, Moderna, and AstraZeneca vaccines differ. Gozzi et al. explored diverse vaccination rollout speeds, prioritization strategies, and vaccine efficacy in six countries worldwide$:$ Egypt, Peru, Serbia, Ukraine, Canada, and Italy. Accordingly, they sought to elucidate the possible effects of relaxed COVID-safe behavior in response to vaccine rollouts^29^. However, their study was limited because their assumed vaccination effects and rates approximated reality. We incorporated real data on vaccine doses across all age groups into our model instead of assuming the vaccination rates (Supplementary Table 2). Third, we did not deliberate on the vaccination expenses and social costs generated by the implementation of NPIs because of the paucity of such data. The existing studies have qualitatively computed such costs by using a mathematical model and adjusting parameters depending on policy decisions^5, 27^.

Notwithstanding its limitations, the present study offers valuable insights into the cost-effectiveness of interventions against COVID-19 variants across different age groups. We approximated that the daily cost per person ranged similarly between $10 and $35 for both the Delta and Omicron variants. However, the cost drivers differed: Omicron’s higher costs resulted from the upsurge in COVID-19 infections, while Delta’s higher costs stemmed from increased deaths because of the higher fatality rate. This finding highlights the importance of conducting cost-effect analyzes for both infections and fatalities in evaluating the impact of COVID-19 variants.

Our findings can help policymakers allocate resources more effectively and tailor strategies to specific age groups and circumstances to ultimately generate improved public health outcomes. Despite the stated drawbacks, we believe that our method delivers a useful approach that can inform health-related policies. Our study’s assessment of the costs associated with reducing infections and deaths across various age groups as well as its consideration of the impact of different variants can benefit and guide policymakers.

## Methods

We constructed an age-structured model in this study to describe the dynamics related to COVID-19 transmissions, evaluate the effectiveness of vaccination, and determine the impact of variants. We also analyzed the cost-effectiveness of the Delta and Omicron variants.

**Figure 4:**
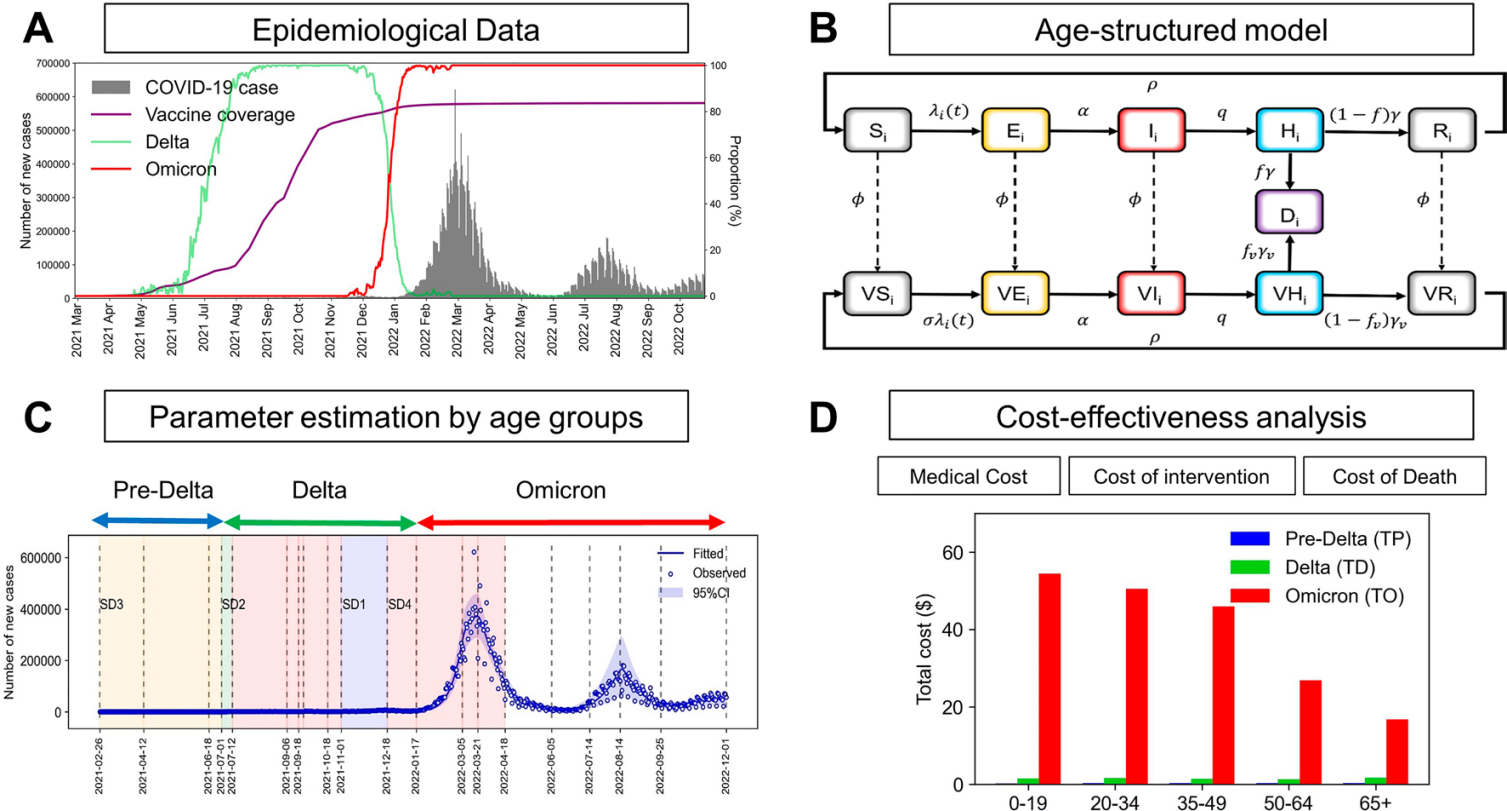
Model components. (**A**) COVID-19 data used in this study for the Republic of Korea. (**B**) Age-structured SEIHR model with contact patterns incorporating the effect of control interventions and variants. (**C**) The compartment model was used to estimate the time-varying transmission and fatality rates. (**D**) The cost-effectiveness analysis based on age groups of COVID-19 cases, and deaths according to variants.

The mathematical model incorporating the contact pattern within various age groups was fitted using the behavioral modification and epidemiological data acquired in the Republic of Korea between February 26, 2021, and November 30, 2022. The parameters were accordingly estimated from the model fit to derive age-stratified COVID-19 confirmation data ($n=\mathrm{27,013,570}$) and fatalities ($n=\mathrm{13,117}$) (refer to Fig. 4A,B). We then evaluated the impact of the Delta and Omicron variants by dividing time intervals into periods labeled, for instance, pre-Delta, Delta, and Omicron. Figure 4B,C illustrate that we qualitatively computed the costs of COVID-19 in terms of medical, intervention, and death-related expenses.

### Data

We acquired epidemiological data spanning February 2021 to December 2022 from the Korea Disease Control and Prevention Agency (KDCA)^30^. We excluded imported cases and attended to the characteristics of locally transmitted cases to examine the number of daily reported COVID-19 cases and related deaths in the Republic of Korea, as summarized in Supplementary Tables 3 and 4. Age-specific data relating to confirmed cases as well as the number of vaccinated individuals were classified into five age groups: 0–19, 20–34, 35–49, 50–64, and 65+ years. We obtained the demographic composition of Korean residents for February 2021 from the Korean Statistical Information Service (KOSIS)^31^. We ascertained the number of citizens who had received the second dose of the COVID-19 vaccine from KDCA but did not take specific types of vaccines into account. We assumed that people were vaccinated equivalently for a week to generate the daily number of vaccine doses. We used population size as reported in 2021. However, we encountered the issue that the total vaccinations in 2021 and 2022 exceeded the population size in some age groups. We adjusted the 2022 vaccination proportions uniformly for each age group to address this problem. As summarized in Supplementary Table 2, this adjustment ensured that the cumulative vaccinations up to 2022 did not surpass the population and maintained the overall vaccination figures within appropriate limits. Supplementary Fig. 2 presents the transmission dynamics of the epidemiological data, including age-stratified cases, deaths, and vaccination coverage. The baseline (pre-pandemic) contact matrixes for Korea were extracted from a previous study conducted by Prem et al.^32^. Supplementary Table 5 demonstrates that the contact matrix after the COVID-19 pandemic was inferred based on the attendance rate of schools^33^. We classified the time intervals into 19 periods in accordance with significant features related to the transmission dynamics and the major events of varied interventions by the Korean government, as listed in Supplementary Table 6.

### Age-structured transmission model

**Figure 5:**
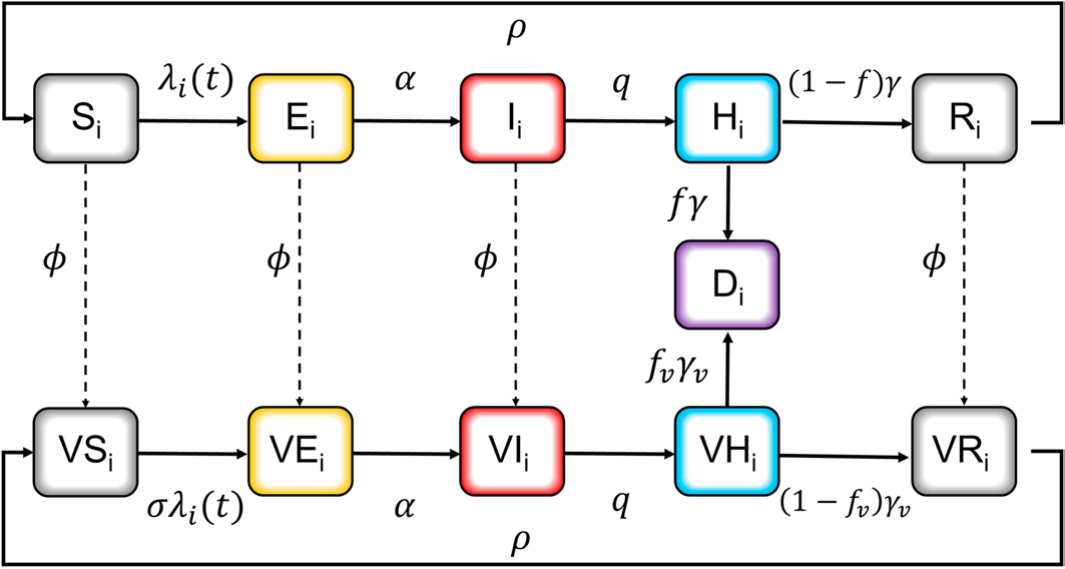
Age-structured compartmental model.

Given the above-stated data, we proposed an age-structured compartmental model as displayed in Fig. 5, including deaths as reported by Tenforde et al.^10^. Figure 4 illustrates a schematic of the transmission model with the population stratified into five age groups, in which set $A=$(0–19, 20–34, 35–49, 50–64, 65+) (i.e., ${n}_{a}=5$). In principle, the developed model was structured as susceptible-exposed-infectious-confirmed-recovered or dead. In this framework, susceptible individuals ($S$) may become infected but not yet infectious ($E$) through contact with infectious individuals ($I$). Individuals were classified as $I$ when they became infectious, including both symptomatic and asymptomatic cases. Imported cases were not considered because of the extensive number of individuals infected through local transmissions. We overlooked under-reported cases because the model fit was identified for reported cases. Infectious individuals were confirmed and reported ($H$). Individuals confirmed to have contracted COVID-19 either recovered ($R$) or died ($D$) from the disease. The intensity of the infection was defined by a weighted sum of the fraction of the infectious population in different age groups. We did not include natural birth and death processes in this measure because we inspected the transmission dynamics occurring within a year.

We assumed based on each individual’s disease record that vaccines could be independently delivered to all persons, excluding those who were currently confirmed ($\mathrm{classified as }H$). People who had received two vaccine doses (2nd vaccination) were deemed vaccinated individuals in this study. This assumption reflects that individuals who are currently infectious, including symptomatic cases, are vaccinated as well; however, the proportion of such individuals was low in comparison to the total number of people classified as $I$. Vaccination presumed three mechanisms of action caused by reduced severity: (i) reducing susceptibility (${\sigma }_{S}$); (ii) reducing infectivity (${\sigma }_{I}$); and (iii) reducing fatality rates (${f}_{v}$). Notably, a small fraction of the recovered population lost immunity obtained from vaccination. We assumed that immune protection was achieved immediately from vaccination or infection.

### Model definition

Discrete forces of infection were determined for both unvaccinated and vaccinated individuals. Supplementary Table 7 offers a detailed description of the model parameters. The model was implemented using a system of ordinary differential equations displayed in Supplementary Information A. The transmission model was stratified into ${n}_{a}=5$ age groups: 0–19, 20–34, 35–49, 50–64, and 65+ years. ${D}_{i}$ indicated disease-induced deaths. The number of unvaccinated individuals in the age group $i$, $i = 1, \ldots , n_{a}$, who were susceptible (${S}_{i}$), exposed (${E}_{i}$), infectious (${I}_{i}$), confirmed and reported (${H}_{i}$), and recovered (${R}_{i}$) were expressed through Eqs. (1)–(5). Similarly, the number of vaccinated individuals in the age group $i$, who were vaccinated susceptible (${VS}_{i}$), exposed (${VE}_{i}$), infectious (${VI}_{i}$), confirmed and reported (${VH}_{i}$), and recovered (${VR}_{i}$) were expressed through Eqs. (6)–(10). Newly reported cases were defined as ${h}_{i}(t)=q{I}_{i}(t)+q{VI}_{i}(t)$.

### Effective reproduction number

The basic reproduction number denoted by ${R}_{0}$ was defined as the average number of secondary cases triggered by an infected individual in a completely susceptible population. This index indicates the influence of control interventions, i.e., active disease transmission continues (${R}_{0}>1$) or ceases (${R}_{0}<1$)^34^. Thus, ${R}_{0}<1$ must be sustained to prevent an outbreak. The entire population ceases to be susceptible when a disease has already spread and control interventions are implemented. Therefore, the effective reproduction number, ${R}_{e}(t)$, varies temporally and quantifiably represents the instantaneous transmissibility of the disease. The effective control of a disease corresponds to ${R}_{e}(t)<1$, as described in^35^. Accordingly, we derived ${R}_{0}$ and ${R}_{e}(t)$ for Eqs. (1)–(11) in Supplementary Information B.

### Time-varying contact matrix

We incorporated the contact matrixes reported by^32^, in which age- and location-specific contact matrixes were projected for 16 age groups across 152 countries. $M$ represents the contact matrix, described in detail in Supplementary Information C. The country-specific contact matrixes with 15 age groups (pre-pandemic) are reported by^32^. The contact matrix characterized the degree of contact between the age groups, which was expressed as the linear combination of contacts in households ($H$), workplaces ($W$), schools ($S$), and other locations ($O$). Therefore, the contact matrix $M$ was defined as follows.

$$M = c_{H} M_{H} + c_{W} M_{W} + c_{S} M_{S} + c_{O} M_{O} ,$$

where the coefficients were designated as $C=({c}_{H},{c}_{W},{c}_{S},{c}_{O})$. Social interactions were denoted via age group-to-age group contact matrixes, which could be modified using available data. Thus, we constructed a contact matrix with five age groups from the baseline contact matrixes, as described in Supplementary Information C. Supplementary Fig. 3 exhibits every contact matrix. The contact patterns were influenced by the reinforcement or relaxation of control interventions such as social distancing, as summarized in Supplementary Table 6. Therefore, the time-varying contact matrixes for periods ${P}_{1}-{P}_{19}$ are presented in Supplementary Information C. We incorporated the effects of social distancing on contact matrixes by varying the coefficients $C=({c}_{H},{c}_{W},{c}_{S},{c}_{O})$ as listed in Supplementary Table 8.

### Estimation of parameters

The initial condition was considered as values simulated for February 26, 2021, by fitting the model from July 16, 2020, after the beginning of the second wave^36^. Supplementary Table 9 presents the initial condition, where $t=0$ indicates February 26, 2021. We fitted the model to COVID-19 cases and deaths reported from February 26, 2021, to November 30, 2022, classifying 19 time intervals ($n=19$) as described in Supplementary Table 6.

This study aimed to minimize the variations between (1) the estimated (${h}_{i}(t)=q{I}_{i}(t)+q{VI}_{i}(t)$) and observed (${OI}_{i}(t)$) cases of COVID-19, and (2) the estimated (${d}_{i}(t)=\gamma {f}_{i}{H}_{i}(t)+{\gamma }_{v}{f}_{v,i}{VH}_{i}(t)$), and observed (${OD}_{i}(t)$) COVID-19-related deaths. The objective function ${J}_{k}$ during time $t$ with $t \in P_{k}$, $t = \left\{ {1,2, \ldots ,n_{k} } \right\}$ was defined as follows.

$${J}_{k}=\sum_{t=1}^{{n}_{k}}\sum_{i=1}^{{n}_{a}}\left(({{OI}_{i}(t)-{h}_{i}(t))}^{2}+({{OD}_{i}(t)-{d}_{i}(t))}^{2}\right)/{n}_{k}$$

We estimated the transmission rate (${b}_{i}$) and death rate (${f}_{i}$) for each period $P_{k}$, $k = 1,2, \ldots ,19$ in the age group $i$ using the least-squared method (lsqcurvefit of MATLAB-embedded function) by minimizing the objective function ${J}_{k}$. We evaluated 95%-confidence intervals using 1000 samples from a normal distribution.

### Cost-effectiveness analysis across age groups and variants

#### Definition of cost

We investigated the social cost of COVID-19-related infections and deaths during the projection period (i.e., four weeks) apart from the cost of accompanying NPIs and vaccinations. The cost required for each age group was evaluated for three types of individuals: confirmed cases, close contacts, and deaths. The cost was defined as the social outlay per person per day.

Overall, the cost encompassed medical treatment $({\mathrm{Medical cost}}_{i}$), intervention (${\mathrm{Intervention cost}}_{i}$), and death (${\mathrm{Death cost}}_{i}$). Table 2 describes the definition of vital cost-related factors in the age-structured model. We calculated the total cost for three targeted populations by age group: (i) reported cases, (ii) individuals coming into close contact with infected cases (close contact), and (iii) deaths because of COVID-19. The social cost comprised the outlays necessitated by medical treatment, interventions, and death.

**Table 2:** Components of cost by age group.

Cost	Description
Medical cost	The medical costs comprise the hospitalization cost and non-hospitalization cost required to treat an actual confirmed case for each age group *i* ${\mathrm{Medical cost}}_{i}(t)=(Hospitalization cost+non-hospitalization\mathrm{ cost}) {h}_{i}(t)$
Cost of intervention	The intervention cost represents the loss cost caused by the COVID-19 prevention efforts, such as the personal or societal cost of social distancing for confirmed cases and closed contacts with confirmed cases ${\mathrm{Intervention cost}}_{i}(t)=\kappa \frac{\sum_{j=1}^{{n}_{a}}{m}_{i,j}}{q}\frac{{\mathrm{Income}}_{i}}{\gamma } {h}_{i}(t)$
Cost of death	The cost of death with age group $i$ in time $t$ was calculated by multiplying VSL with the remaining life expectancy for deaths ${\mathrm{Death cost}}_{i}(\mathrm{t})=\upvarepsilon {\mathrm{VSL}}_{USA}\frac{{GDP}_{Korea}}{{GDP}_{USA}}{d}_{i}(t)$

First, medical costs comprised hospitalization and non-hospitalization expenses incurred for the treatment of an actual confirmed case. Hospitalization costs included the amount required for diagnostic testing for COVID-19, treating a confirmed case, and so on. Non-hospitalization costs involved the amount spent for epidemiological investigation, data construction, childcare, and household labor for an infected case^24^. ${\mathrm{Medical cost}}_{i}$ in time $t$ for an infected case in age group $i$ was calculated as the medical cost for an infected case multiplied by the estimated number of confirmed cases (${h}_{i}(t)$). Second, intervention cost represented the losses incurred because of COVID-19 prevention efforts, for instance, personal or societal outlays accruing to confirmed cases and their close contacts because of social distancing. ${\mathrm{Intervention cost}}_{i}$ in time $t$ was evaluated by multiplying the total number of close contacts during the infectious period for every new confirmed case in age group $i$ ($\sum_{j=1}^{{n}_{a}}{m}_{i,j}/q$) and accounting for the average income of age group $i$ during the isolation period (${\mathrm{Income}}_{i}/\gamma$), the rate of labor losses during the isolation period ($\kappa$), and the estimated number of confirmed cases (${h}_{i}(t)$). Third, the value of a statistical life (VSL) indicates the statistical death cost as dollar units per statistical death during life expectancy^37^. The cost of death for age group $i$ in time $t$ was calculated by multiplying VSL with the remaining life expectancy for those who died of COVID-19. All cost-related parameters are summarized in Table 3.

**Table 3:** Description of cost parameters.

Cost	Notation	Cost (unit)	0–19	20–34	35–49	50–64	65+	Refs.
Medical cost	–	Hospitalization($/person)	3738					^38^
	–	Non-hospitalizations($/person)	3583					^38^
Cost of intervention	$$\kappa$$	Labor loss rate ($${\text{day}}^{-1}$$)	0.3					Assumed
	$$1/q$$	Period between infectiousness and hospitalization (day)	6.8					^39^
	$$\gamma$$	Recovered/removed rate ($${\text{day}}^{-1}$$)	1/10					^40^
	$${\mathrm{Income}}_{i}$$	Average income for age group ($/day)	23	68	104	90	95	^41^
Cost of death	$$\upvarepsilon$$	Rate of remained life expectancy (year)	0.878	0.676	0.493	0.318	0.108	^42^
	$${\text{VSL}}_{\text{USA}}$$	VSL in USA ($)	9,631,000					^37^
	$${\text{GDP}}_{\text{Korea}}$$	GDP per capita in Korea ($)	30,943					^43^
	$${\text{GDP}}_{\text{USA}}$$	GDP per capita in USA ($)	62,683					^37^

Rate of remained life expectancy calculated as average life expectancy-median of age groups Average life expectancy. VSL indicates the value of a statistical life. The exchange rate is considered as 1200 won per dollar.

#### Evaluating variant-specific cost changes across age groups

We defined the time point at which a variant became dominant as when it accounted for more than 50% of new infections. We categorized three time phases based on this definition: pre-Delta variant from February to June 2021 (TP), Delta variant from July to December 2021 (TD), and Omicron variant from January to November 2022 (TO). We analyzed the characteristics of each variant and their effects on different age groups by comparing age-specific average values of ${\varvec{b}}$, ***f****,* and ${R}_{\mathrm{t}}$ during the respective periods of dominance of each variant, where ${\varvec{b}}=({b}_{1}, \dots , {b}_{{\mathrm{n}}_{\mathrm{a}}})$ and ${\varvec{f}}=({f}_{1}, \dots , {f}_{{\mathrm{n}}_{\mathrm{a}}})$.

We scrutinized the changes in the three costs related to medical treatment, intervention, and death for the fitting period from February 26, 2021, to November 30, 2022, as well as the variant-specific average costs for each age group. However, absolute comparisons were rendered difficult because of the differences in the number of COVID-19 cases, deaths, and vaccination rates vis-à-vis variants during the COVID-19 pandemic.

We aimed to forecast the potential costs associated with a new variant exhibiting characteristics similar to the pre-Delta, Delta, and Omicron strains. First, we estimated the daily average costs per capita from December 1, 2022, to December 31, 2022, assuming the average transmission rate ($\overline{{\varvec{b}} }$) and case-fatality rate ($\overline{{\varvec{f}} }$) from the three previously defined phases TP, TD, and TO, as shown in Table 1. Second, the number of COVID-19 cases and deaths was estimated for one month (from December 1 to 31, 2022) using $\overline{{\varvec{b}} }$ and $\overline{{\varvec{f}} }$ according to the emergence of the pre-Delta, Delta, and Omicron variants. Third, we compared the cost patterns of the emergence of a new variant. First, we conducted a sensitivity analysis based on the 65+ age group to reveal costs when the transmission rate (${b}_{5}$) ranged from 0.01 to 0.5 and the case-fatality rate (${f}_{5}$) ranged from 0.001 to 0.1. Transmission rates for other age groups were calculated based on the ratios of age-specific transmission rates estimated in ${P}_{19}$. The case-fatality rates for other age groups were also computed using the same approach.

### Ethical approval

This study analyzed data that are publicly available through previous reports^3^. The datasets used in this study were summarized and anonymized. Ethical approval is not required for analysis of publicly available data with no identifying information.

### Supplementary Information

Supplementary Information.

## Data Availability

The datasets used and/or analyzed during the current study available from the corresponding author on reasonable request.
