# Author Correction: Cost-effectiveness analysis of COVID-19 variants effects in an age-structured model

**DOI:** 10.1038/s41598-024-54225-3

**Published:** 2024-02-14

**Authors:** Giphil Cho, Young Jin Kim, Sang‑hyup Seo, Geunsoo Jang, Hyojung Lee

**Affiliations:** 1 Department of Artificial Intelligence and Software, Kangwon National University, Chuncheon, Gangwon 25913 Republic of Korea; 2 Division of Data Analysis, Center for Global R&D Data Analysis, Korea Institute of Science and Technology Information (KISTI), Seoul, 02456 Republic of Korea; 3 National Institute for Mathematical Sciences, Daejeon, 34047 Republic of Korea; 4 Nonlinear Dynamics and Mathematical Application Center, Kyungpook National University, Daegu, 41566 Republic of Korea; 5 Department of Statistics, Kyungpook National University, Daegu, 41566 Republic of Korea

Correction to: *Scientific Reports* 10.1038/s41598-023-41876-x, published online 22 September 2023

The original version of this Article contained an error in the Funding section, where an NRF grant number was incorrectly stated for H. Lee.

“H. Lee was supported by the National Research Foundation of Korea (NRF) grant funded by the Korea government (MSIT) (Nos. NRF-2022R1A2C3011711, NRF-2022R1A5A1033624).”

now reads:

“H. Lee was supported by the National Research Foundation of Korea (NRF) grant funded by the Korea government (MSIT) (No. NRF-2022R1C1C1006237, NRF-2022R1A5A1033624).”

The original Article has been corrected.

