# Supplementary material for: Cost-effectiveness analysis of COVID-19 variants effects in an age-structured model
Source: Sci Rep. 2023 Sep 22;13:15844. doi: 10.1038/s41598-023-41876-x (PMC10516971; doi:10.1038/s41598-023-41876-x)
Supplement: Supplementary file 1 — Supplementary Information. [file 41598_2023_41876_MOESM1_ESM.pdf]

# Supplementary Information

# Cost-Effectiveness Analysis of COVID-19 Variants Effects in an Age-Structured Model

Giphil Cho[1], Young Jin Kim[2], Sang-hyup Seo[3], Geunsoo Jang[4] and Hyojung Lee[5*]

[1]Department of Artificial Intelligence and Software, Kangwon National University, Gangwon, 25913, Republic of Korea

[2]Center for Global R&D Data Analysis, Division of Data Analysis, Korea Institute of Science and Technology Information (KISTI), Seoul, 02456, Republic of Korea

[3]National Institute for Mathematical Sciences, Daejeon, 34047, Republic of Korea

[4]Nonlinear Dynamics and Mathematical Application Center, Kyungpook National University, Daegu, 41566, Republic of Korea

[5]Department of Statistics, Kyungpook National University, Daegu, 41566, Republic of Korea

*Corresponding author

Hyojung Lee

Department of Statistics, Kyungpook National University, Daegu, 41566, Republic of Korea

E-mail: hjlee@knu.ac.kr

## A. Age-structured compartmental model

The transmission model was stratified into $n_a = 5$ age groups: 0–19, 20–34, 35–49, 50–64, and 65+ years. The numbers of unvaccinated individuals in age group $i$, $i = 1, \cdots, n_a$, who were susceptible ($S_i$), exposed ($E_i$), infectious ($I_i$), confirmed and reported ($H_i$), and recovered ($R_i$) were expressed in Equations (1)–(5). Similarly, the numbers of vaccinated individuals in age group $i$, who were vaccinated but susceptible ($VS_i$), exposed ($VE_i$), infectious ($VI_i$), confirmed and reported ($VH_i$), and recovered ($VR_i$) were expressed in Equations (6)–(10), $D_i$

indicated disease-induced deaths in Equation (11) as follows:

$$\frac{dS_i}{dt} = -\lambda_i S_i - \phi_i(t)\frac{S_i}{S_i + E_i + I_i + R_i} + \rho R_i \tag{1}$$

$$\frac{dE_i}{dt} = \lambda_i S_i - \alpha E_i - \phi_i(t)\frac{E_i}{S_i + E_i + I_i + R_i} \tag{2}$$

$$\frac{dI_i}{dt} = \alpha E_i - qI_i - \phi_i(t)\frac{I_i}{S_i + E_i + I_i + R_i} \tag{3}$$

$$\frac{dH_i}{dt} = qI_i - \gamma H_i \tag{4}$$

$$\frac{dR_i}{dt} = \gamma(1-f_i)H_i - \phi_i(t)\frac{R_i}{S_i + E_i + I_i + R_i} - \rho R_i \tag{5}$$

$$\frac{dVS_i}{dt} = -\sigma_S \lambda_i VS_i + \phi_i(t)\frac{S_i}{S_i + E_i + I_i + R_i} + \rho VR_i \tag{6}$$

$$\frac{dVE_i}{dt} = \sigma_S \lambda_i VS_i - \alpha VE_i + \phi_i(t)\frac{E_i}{S_i + E_i + I_i + R_i} \tag{7}$$

$$\frac{dVI_i}{dt} = \alpha VE_i - qVI_i + \phi_i(t)\frac{I_i}{S_i + E_i + I_i + R_i} \tag{8}$$

$$\frac{dVH_i}{dt} = qVI_i - \gamma_v VH_i \tag{9}$$

$$\frac{dVR_i}{dt} = \gamma_v\left(1-f_{v,i}\right)VH_i + \phi_i(t)\frac{R_i}{S_i + E_i + I_i + R_i} - \rho VR_i \tag{10}$$

$$\frac{dD_i}{dt} = \gamma f_i H_i + \gamma_v f_{v,i} VH_i \tag{11}$$

where $m_{ij}$ denotes the element of the contact matrix, $M$. $m_{ij}$ signifies the contact rate of an individual in the age group $i$ with individuals in the age group $j$ (i.e. $M = [m_{ij}]$). The intensity of infection for the age group $i$ is expressed as

$$\lambda_i(t) = \sum_{k=1}^{n} \frac{b_i m_{i,k}(I_k + \sigma_I VI_k)}{N_i(t)}$$

where $N_i$ denotes the number of individuals in the age group $i$, $N_i(t) = S_i(t) + E_i(t) + I_i(t) + H_i(t) + R_i(t) + VS_i(t) + VE_i(t) + VI_i(t) + VH_i(t) + VR_i(t)$.

## B. Effective reproduction number

For the calculation of $R_e$ , the disease-free equilibrium can be defined as $x_0 = (S_i, 0,0,0,0, VS_i, 0,0,0,0,0)$ and $\phi_i = 0$.

Thus, $F$ and $V$ are $30 \times 30$ matrixes at $x_0$ expressed as

$$F(x_0) = \begin{bmatrix} 0_{5,5} & A_1 & 0_{5,5} & 0_{5,5} & \sigma_I A_1 & 0_{5,5} \\ 0_{5,5} & 0_{5,5} & 0_{5,5} & 0_{5,5} & 0_{5,5} & 0_{5,5} \\ 0_{5,5} & 0_{5,5} & 0_{5,5} & 0_{5,5} & 0_{5,5} & 0_{5,5} \\ 0_{5,5} & \sigma_S A_2 & 0_{5,5} & 0_{5,5} & \sigma_S \sigma_I A_2 & 0_{5,5} \\ 0_{5,5} & 0_{5,5} & 0_{5,5} & 0_{5,5} & 0_{5,5} & 0_{5,5} \\ 0_{5,5} & 0_{5,5} & 0_{5,5} & 0_{5,5} & 0_{5,5} & 0_{5,5} \end{bmatrix}$$

where $A_1 = \text{diag}\{b_1 S_1, b_2 S_2, \ldots, b_5 S_5\} \cdot M \cdot \text{diag}\left\{\frac{1}{N_1}, \frac{1}{N_2}, \ldots, \frac{1}{N_5}\right\}, A_2 \ \text{diag}\{b_1 VS_1, b_2 VS_2, \ldots, b_5 VS_5\} \cdot M \cdot \text{diag}\left\{\frac{1}{N_1}, \frac{1}{N_2}, \ldots, \frac{1}{N_5}\right\}$, and $M$ represents the contact matrix.

$$V(x_0) = \begin{bmatrix} B & 0_{5,5} & 0_{5,5} & 0_{5,5} & 0_{5,5} & 0_{5,5} \\ -B & C & 0_{5,5} & 0_{5,5} & 0_{5,5} & 0_{5,5} \\ 0_{5,5} & -C & D & 0_{5,5} & 0_{5,5} & 0_{5,5} \\ 0_{5,5} & 0_{5,5} & 0_{5,5} & B & 0_{5,5} & 0_{5,5} \\ 0_{5,5} & 0_{5,5} & 0_{5,5} & -B & C & 0_{5,5} \\ 0_{5,5} & 0_{5,5} & 0_{5,5} & 0_{5,5} & -C & D \end{bmatrix}$$

where $B = \alpha I_5, C = q I_5$, and $D = \gamma I_5$.

Thus, the inverse matrix of $V$ can be derived as

$$V(x_0)^{-1} = \begin{bmatrix} B^{-1} & 0_{5,5} & 0_{5,5} & 0_{5,5} & 0_{5,5} & 0_{5,5} \\ C^{-1} & C^{-1} & 0_{5,5} & 0_{5,5} & 0_{5,5} & 0_{5,5} \\ D^{-1} & D^{-1} & D^{-1} & 0_{5,5} & 0_{5,5} & 0_{5,5} \\ 0_{5,5} & 0_{5,5} & 0_{5,5} & B^{-1} & 0_{5,5} & 0_{5,5} \\ 0_{5,5} & 0_{5,5} & 0_{5,5} & C^{-1} & C^{-1} & 0_{5,5} \\ 0_{5,5} & 0_{5,5} & 0_{5,5} & D^{-1} & D^{-1} & D^{-1} \end{bmatrix}$$

Therefore, the subsequent generation matrix $K$ can be defined as

$$K = FV^{-1} = \begin{bmatrix} \frac{1}{q}A_1 & \frac{1}{q}A_1 & 0_{5,5} & \frac{\sigma_I}{q}A_1 & \frac{\sigma_I}{q}A_1 & 0_{5,5} \\ 0_{5,5} & 0_{5,5} & 0_{5,5} & 0_{5,5} & 0_{5,5} & 0_{5,5} \\ 0_{5,5} & 0_{5,5} & 0_{5,5} & 0_{5,5} & 0_{5,5} & 0_{5,5} \\ \frac{\sigma_S}{q}A_2 & \frac{\sigma_S}{q}A_2 & 0_{5,5} & \frac{\sigma_S\sigma_I}{q}A_2 & \frac{\sigma_S\sigma_I}{q}A_2 & 0_{5,5} \\ 0_{5,5} & 0_{5,5} & 0_{5,5} & 0_{5,5} & 0_{5,5} & 0_{5,5} \\ 0_{5,5} & 0_{5,5} & 0_{5,5} & 0_{5,5} & 0_{5,5} & 0_{5,5} \end{bmatrix}.$$

Ultimately, the effective reproduction number $R_e$ was computed as the spectral radius $\rho(K)$ of the next-generation matrix $K$, i.e. $R_e = \rho(K)$.

# C. Contact matrix

## C.1 Calculations of contact matrixes in South Korea

The contact matrix $M$ was defined as

$$M = (m_{ij}),$$

where each element is $m_{ij}$, denoting the mean number of contacts of an individual in the age group $i$ with individuals in the age group $j$ per day. More specifically, $m_{ij}$ can be defined as

$$m_{ij} = t_{ij}/n_i,$$

where $t_{ij}$ denotes the total number of contacts between individuals in the age group $i$ and age group $j$ per day and $n_i$ is the total number of individuals in $i$.

## C.2 Reciprocity corrections of contact matrixes

This study focused on five age groups based on the classification reported in [1–3] 0–19, 20–34, 35–49, 50–64, and 65+ years. Otherwise, each $16 \times 16$ contact matrix from [4] for communication groups at home, school, work, and others was determined for the age groups specified as 0–4, 5–9, 10–14, 15–19, 20–24, 25–29, 30–34, 35–39, 40–44, 45–49, 50–54, 55–59, 60–64, 65–69, 70–74, and 75+ years.

Thus, we were required to transform the contact matrixes pertaining to 16 age groups to target the contact matrixes for the five age groups established for this study. The Supplementary Information noted in [5] stipulates five requirements for the aptness of the target contact matrix for integration in the transmission model. However, the current and target regions of this study are representative of the entirety of the Republic of Korea, requiring us to satisfy the following requirements: (1) population segmentation into five age groups (i.e. modified age groups), and (2) reciprocity condition, i.e. the symmetry of the total contact matrix $T = (t_{ij})$.

## C.3 Transformation of the contact matrix

Let the current 16 age groups and the $16 \times 16$ contact matrix $\widetilde{M}$ be assumed from [4] for the Republic of Korea. The study's goal was to obtain a suitable $5 \times 5$ contact matrix $M$ for the five target age groups. First, we reconstructed the contact matrix $\widehat{M} = (\widehat{m}_{ij})$, which ensured the symmetry of the total contact matrix of $\widehat{M}$. Supposing $\tilde{n} \in \mathbb{R}^{16}$ denotes the vector with $i$-th element $\tilde{n}_i$ denoting the population of the current age group $i$, and $\mathrm{n} \in \mathbb{R}^5$ represents the vector with $k$-th element $n_k$ representing the population of the target age group $k$. Let $\tilde{t}_{ij} = \widetilde{m}_{ij}\tilde{n}_i$ indicating the total contact number between age groups $i$ and $j$.

We obtained the symmetric total contact matrix $\hat{T} = \frac{1}{2}(\tilde{T} + \tilde{T}^T)$ where $\tilde{T} = (\tilde{t}_{ij})$ upon applying the symmetry transformation. Thereafter, $\widehat{M}$ could be defined as follows:

$$\widehat{m}_{ij} = \hat{t}_{ij}/\tilde{n}_i \tag{1}$$

We obtained the contact matrix $\widehat{M}$ with the total contact matrix $\hat{T}$ ensuring symmetry.

Supplementary Information B in [5] illuminates that the elements of the target contact matrix $M = (m_{kl})$ were obtained based on the following formula:

$$m_{kl} = \frac{1}{n_k} \sum_{i \in G} \sum_{j \in G} \widehat{m}_{ij}\tilde{n}_i \frac{\overline{n}_{jl}}{\tilde{n}_j} \frac{\overline{n}_{ik}}{\tilde{n}_i}, \quad k, l \in G', \tag{2}$$

where $G$ denoted the set of indices of the current age group, $\mathrm{G}'$ signified the set of indices of the target age group, and $\overline{n}_{jl}$ represented the overlapped population for $j \in G$ and $l \in G'$. In equation (2), $\overline{n}_{jl} = n_j$ if the age group $j \in G$ is included in the age group $l \in G'$, otherwise, $\overline{n}_{jl} = 0$.

Thus, we obtained that

$$m_{kl} = \frac{1}{n_k} \sum_{i \in G} \sum_{j \in G} \hat{t}_{ij}\, \xi_{jl}\, \xi_{ik}, \tag{3}$$

where

$$\xi_{jl} = f(x) = \begin{cases} 1, \text{if the group } j \in G \text{ is included in the group } l \in G', \\ 0, \text{otherwise.} \end{cases} \quad (4)$$

To intuitively explain equation (3), we considered the target total contact matrix $T = (t_{kl})$ of the target contact matrix $M$. As $t_{kl} = m_{kl}n_k$, we derived that

$$t_{kl} = \sum_{i \in G} \sum_{j \in G} \hat{t}_{ij}\, \xi_{jl} \xi_{ik}. \quad (5)$$

For instance, $t_{0-9,0-9} = \hat{t}_{0-4,0-4} + \hat{t}_{5-9,0-4} + \hat{t}_{0-4,5-9} + \hat{t}_{5-9,5-9}$. Let $W \in \mathbb{R}^{16\times5}$ be defined as

$$W^{\top} = \begin{bmatrix} 1&1&1&1&0&0&0&0&0&0&0&0&0&0&0&0 \\ 0&0&0&0&1&1&1&0&0&0&0&0&0&0&0&0 \\ 0&0&0&0&0&0&0&1&1&1&0&0&0&0&0&0 \\ 0&0&0&0&0&0&0&0&0&0&1&1&1&0&0&0 \\ 0&0&0&0&0&0&0&0&0&0&0&0&0&1&1&1 \end{bmatrix} \quad (6)$$

Thereafter, we could simplify the above transformation to the following matrix calculation process.

1. $\tilde{T} = \mathrm{diag}(\tilde{n})\widetilde{M}$,
2. $\hat{T} = \frac{1}{2}\left(\tilde{T} + \widetilde{T^{\top}}\right)$,
3. $T = W^{\top}\hat{T}W$,
4. $M = \mathrm{diag}(n)^{-1}T$,

where $\mathrm{diag}(x) = \begin{bmatrix} x_1 & 0 & \cdots & 0 \\ 0 & x_2 & \cdots & 0 \\ \vdots & \vdots & \ddots & \vdots \\ 0 & 0 & \cdots & x_n \end{bmatrix}$, if $x = [x_1, x_2, \cdots, x_n]^{\top}$.

The above transformation enabled the contact matrixes $M_H$, $M_S$, $M_W$, and $M_O$ to be derived for the age groups 0–19, 20–34, 35–49, 50–64, and 65+ years. The matrixes are described in Supplementary Figure 3.

**Supplementary Table 1.** Estimated transmission rates (***b***) and case-fatality rates (***f***) by age groups

| Parameters | Time period | 0–19 | 20–34 | 35–49 | 50–64 | 65+ |
|---|---|---|---|---|---|---|
| | $P_1$ | 0.0093 | 0.0160 | 0.0179 | 0.0301 | 0.0512 |
| | $P_2$ | 0.0074 | 0.0146 | 0.0149 | 0.0232 | 0.0361 |
| | $P_3$ | 0.0115 | 0.0257 | 0.0231 | 0.0228 | 0.0102 |
| | $P_4$ | 0.0117 | 0.0268 | 0.0200 | 0.0290 | 0.0252 |
| | $P_5$ | 0.0123 | 0.0212 | 0.0170 | 0.0224 | 0.0333 |
| | $P_6$ | 0.0125 | 0.0263 | 0.0254 | 0.0304 | 0.1101 |
| | $P_7$ | 0.0000 | 0.0000 | 0.0000 | 0.0071 | 0.0167 |
| | $P_8$ | 0.0121 | 0.0278 | 0.0235 | 0.0491 | 0.1319 |
| | $P_9$ | 0.0142 | 0.0189 | 0.0252 | 0.0783 | 0.2104 |
| ***b*** | $P_{10}$ | 0.0157 | 0.0404 | 0.0425 | 0.1118 | 0.2661 |
| | $P_{11}$ | 0.0129 | 0.0432 | 0.0328 | 0.0415 | 0.0222 |
| | $P_{12}$ | 0.0430 | 0.1366 | 0.1088 | 0.1588 | 0.2763 |
| | $P_{13}$ | 0.0313 | 0.0791 | 0.0655 | 0.1066 | 0.2305 |
| | $P_{14}$ | 0.0195 | 0.0491 | 0.0464 | 0.0640 | 0.1347 |
| | $P_{15}$ | 0.0134 | 0.0463 | 0.0292 | 0.0475 | 0.0956 |
| | $P_{16}$ | 0.0461 | 0.1480 | 0.0995 | 0.1302 | 0.1850 |
| | $P_{17}$ | 0.0423 | 0.1480 | 0.1080 | 0.1803 | 0.3595 |
| | $P_{18}$ | 0.0217 | 0.0453 | 0.0417 | 0.0671 | 0.1659 |
| | $P_{19}$ | 0.0325 | 0.1019 | 0.0834 | 0.1393 | 0.3245 |
| | $P_1$ | 0.0000 | 0.0004 | 0.0008 | 0.0025 | 0.0412 |
| | $P_2$ | 0.0000 | 0.0000 | 0.0001 | 0.0022 | 0.0350 |
| | $P_3$ | 0.0000 | 0.0000 | 0.0003 | 0.0018 | 0.0197 |
| | $P_4$ | 0.0000 | 0.0000 | 0.0005 | 0.0068 | 0.0330 |
| | $P_5$ | 0.0000 | 0.0002 | 0.0010 | 0.0056 | 0.0378 |
| | $P_6$ | 0.0000 | 0.0007 | 0.0005 | 0.0036 | 0.0648 |
| | $P_7$ | 0.0000 | 0.0000 | 0.0018 | 0.0018 | 0.0402 |
| | $P_8$ | 0.0000 | 0.0003 | 0.0014 | 0.0055 | 0.0578 |
| | $P_9$ | 0.0000 | 0.0000 | 0.0000 | 0.0064 | 0.0668 |
| ***f*** | $P_{10}$ | 0.0001 | 0.0002 | 0.0007 | 0.0078 | 0.0752 |
| | $P_{11}$ | 0.0000 | 0.0002 | 0.0016 | 0.0048 | 0.0548 |
| | $P_{12}$ | 0.0000 | 0.0000 | 0.0001 | 0.0006 | 0.0124 |
| | $P_{13}$ | 0.0000 | 0.0000 | 0.0000 | 0.0002 | 0.0022 |
| | $P_{14}$ | 0.0000 | 0.0000 | 0.0000 | 0.0001 | 0.0009 |
| | $P_{15}$ | 0.0000 | 0.0000 | 0.0001 | 0.0004 | 0.0045 |
| | $P_{16}$ | 0.0001 | 0.0000 | 0.0003 | 0.0006 | 0.0086 |
| | $P_{17}$ | 0.0000 | 0.0000 | 0.0000 | 0.0001 | 0.0018 |
| | $P_{18}$ | 0.0000 | 0.0000 | 0.0000 | 0.0002 | 0.0025 |
| | $P_{19}$ | 0.0000 | 0.0000 | 0.0000 | 0.0003 | 0.0036 |

**Supplementary Table 2.** Monthly numbers of the second vaccination against COVID-19 received by age groups from March 2021 to December 2022

| | | Total | 0–19 | 20–34 | 35–49 | 50–64 | 65+ |
|---|---|---|---|---|---|---|---|
| Vaccination | Mar. | 34,357 | 147 | 13,558 | 10,146 | 7,407 | 3,098 |
| | Apr. | 226,437 | 718 | 31,136 | 25,773 | 21,250 | 147,560 |
| | May. | 2,005,304 | 2,162 | 159,199 | 177,444 | 262,626 | 1,403,873 |
| | Jun. | 1,771,269 | 4,046 | 165,830 | 79,531 | 92,152 | 1,429,710 |
| | Jul. | 2,162,768 | 10,964 | 720,541 | 405,459 | 538,792 | 487,012 |
| | Aug. | 8,761,501 | 454,220 | 454,416 | 1,037,582 | 3,096,308 | 3,718,975 |
| | Sep. | 9,734,005 | 139,752 | 1,535,015 | 1,802,508 | 5,576,139 | 680,591 |
| | Oct. | 12,794,393 | 274,734 | 4,736,096 | 5,459,755 | 2,071,292 | 252,515 |
| | Nov. | 2,288,733 | 731,220 | 686,639 | 561,484 | 231,445 | 77,946 |
| | Dec. | 1,368,877 | 701,906 | 304,542 | 207,965 | 99,614 | 54,850 |
| | Jan. | 1,557,009 | 708,211 | 290,054 | 274,926 | 146,296 | 137,523 |
| | Feb. | 322,832 | 130,620 | 62,432 | 61,033 | 34,196 | 34,551 |
| | Mar. | 117,448 | 27,847 | 21,732 | 28,404 | 17,876 | 21,589 |
| | Apr. | 57,444 | 8,356 | 9,753 | 14,027 | 10,436 | 14,871 |
| | May. | 40,164 | 20,491 | 4,506 | 5,020 | 3,533 | 6,614 |
| | Jun. | 37,298 | 27,928 | 2,536 | 2,429 | 1,475 | 2,931 |
| | Jul. | 17,814 | 9,436 | 2,156 | 2,156 | 1,384 | 2,682 |
| | Aug. | 12,331 | 4,360 | 2,125 | 2,040 | 1,281 | 2,525 |
| | Sep. | 10,440 | 4,263 | 1,792 | 1,740 | 925 | 1,719 |
| | Oct. | 10,122 | 5,169 | 1,369 | 1,434 | 783 | 1,366 |
| | Nov. | 7,142 | 2,623 | 1,142 | 1,130 | 693 | 1,553 |
| | Dec. | 7,450 | 2,246 | 1,184 | 1,203 | 811 | 2,007 |

**Supplementary Table 3.** Monthly number of COVID-19 cases by age group from March 2021 to December 2022

| | | Total | 0–19 | 20–34 | 35–49 | 50–64 | 65+ |
|---|---|---|---|---|---|---|---|
| Cases | Mar. | 13,415 | 1,728 | 2,942 | 3,262 | 3,481 | 2,002 |
| | Apr. | 18,927 | 2,433 | 4,038 | 4,354 | 5,255 | 2,847 |
| | May. | 18,331 | 2,380 | 4,353 | 4,439 | 4,714 | 2,445 |
| | Jun. | 16,623 | 2,305 | 4,402 | 4,527 | 4,067 | 1,322 |
| | Jul. | 41,374 | 6,814 | 13,445 | 10,721 | 8,496 | 1,898 |
| | Aug. | 53,077 | 8,965 | 16,795 | 12,722 | 10,938 | 3,657 |
| | Sep. | 59,859 | 9,838 | 19,405 | 15,580 | 10,243 | 4,793 |
| | Oct. | 53,421 | 11,063 | 13,517 | 12,400 | 9,338 | 7,103 |
| | Nov. | 82,527 | 16,743 | 12,243 | 15,157 | 18,820 | 19,564 |
| | Dec. | 183,605 | 40,280 | 29,026 | 36,856 | 42,772 | 34,671 |
| | Jan. | 214,861 | 61,351 | 59,181 | 50,391 | 30,452 | 13,486 |
| | Feb. | 2,288,804 | 627,576 | 558,633 | 554,614 | 362,750 | 185,231 |
| | Mar. | 9,961,604 | 2,632,379 | 2,073,798 | 2,313,962 | 1,774,978 | 1,166,487 |
| | Apr. | 4,142,864 | 930,424 | 840,823 | 955,699 | 811,853 | 604,065 |
| | May. | 866,096 | 183,235 | 210,213 | 187,463 | 166,167 | 119,018 |
| | Jun. | 255,914 | 52,879 | 75,917 | 55,876 | 45,877 | 25,365 |
| | Jul. | 1,416,816 | 326,115 | 374,700 | 316,788 | 254,411 | 144,802 |
| | Aug. | 3,470,972 | 668,008 | 783,572 | 749,483 | 731,974 | 537,935 |
| | Sep. | 1,523,168 | 322,258 | 298,494 | 324,397 | 313,285 | 264,734 |
| | Oct. | 788,619 | 148,364 | 157,529 | 168,814 | 168,437 | 145,475 |
| | Nov. | 1,541,536 | 272,435 | 320,538 | 334,760 | 331,318 | 282,485 |
| | Dec. | 1,960,937 | 346,905 | 424,753 | 442,058 | 423,541 | 323,680 |

**Supplementary Table 4.** Monthly number of COVID-19-related deaths by age group from March 2021 to December 2022

| | | Total | 0–19 | 20–34 | 35–49 | 50–64 | 65+ |
|---|---|---|---|---|---|---|---|
| Death | Mar. | 94 | 0 | 2 | 2 | 9 | 81 |
| | Apr. | 101 | 0 | 0 | 0 | 9 | 92 |
| | May. | 103 | 0 | 0 | 2 | 13 | 88 |
| | Jun. | 65 | 0 | 0 | 0 | 8 | 57 |
| | Jul. | 104 | 0 | 1 | 8 | 42 | 53 |
| | Aug. | 217 | 0 | 4 | 11 | 61 | 141 |
| | Sep. | 197 | 0 | 5 | 14 | 38 | 140 |
| | Oct. | 381 | 0 | 4 | 11 | 46 | 320 |
| | Nov. | 954 | 0 | 1 | 4 | 100 | 849 |
| | Dec. | 2,108 | 3 | 3 | 40 | 201 | 1,861 |
| | Jan. | 798 | 0 | 5 | 19 | 85 | 689 |
| | Feb. | 674 | 3 | 1 | 13 | 56 | 601 |
| | Mar. | 1,740 | 6 | 21 | 32 | 150 | 1,531 |
| | Apr. | 1,517 | 4 | 5 | 28 | 175 | 1,305 |
| | May. | 744 | 2 | 8 | 24 | 79 | 631 |
| | Jun. | 215 | 3 | 1 | 12 | 26 | 173 |
| | Jul. | 244 | 7 | 3 | 8 | 24 | 202 |
| | Aug. | 895 | 4 | 4 | 18 | 82 | 787 |
| | Sep. | 795 | 1 | 6 | 17 | 64 | 707 |
| | Oct. | 514 | 1 | 1 | 7 | 48 | 457 |
| | Nov. | 632 | 0 | 1 | 10 | 53 | 568 |
| | Dec. | 833 | 3 | 4 | 17 | 62 | 747 |

**Supplementary Table 5.** Monthly attendance rate of schools in 2021

| Month | Feb. | Mar. | Apr. | May | Jun | Jul. | Aug. | Sep. | Oct. | Nov. | Dec. |
|---|---|---|---|---|---|---|---|---|---|---|---|
| Rate | 73.90 | 74.00 | 71.87 | 70.91 | 77.86 | 49.58 | 27.97 | 70.80 | 82.50 | 83.36 | 88.65 |

The average attendance rates of elementary, middle-, and high-school students for almost a week; data sourced from the Ministry of Education of the Republic of Korea [6].

**Supplementary Table 6.** Major events of varied interventions implemented by the Korean government in the established periods [7]

| Period | Date | Description |
|---|---|---|
| $P_1$ | 26 Feb. 2021– 11Apr. 2021 | Vaccination against to COVID-19 starts |
| $P_2$ | 12 Apr. – 17 Jun. | Maintained to Level 2 of SD in Seoul metropolitan area (SMA), enhanced intervention in high risk region, gatherings banned at entertainment facilities |
| $P_3$ | 18 Jun. – 30 Jun. | Rapidly spreading delta variant |
| $P_4$ | 01 Jul. – 11 Jul. | Implementation of changed control policy of SD with Level 2 in SMA |
| $P_5$ | 12 Jul. – 05 Sep. | Enhanced to Level 4 in SMA |
| $P_6$ | 06 Sep. – 17 Sep. | Restricted gatherings events to no more than 6 people, including two vaccinated people in SMA, relaxed to school closure |
| $P_7$ | 18 Sep. – 22 Sep. | Start of Thanksgiving Day |
| $P_8$ | 23 Sep. – 17 Oct. | End of Thanksgiving Day |
| $P_9$ | 18 Oct. – 31 Oct. | Restricted gathering events to no more than 8 people, including four vaccinated people in SMA |
| $P_{10}$ | 01 Nov. – 17 Dec. | Disease prevention measures and restrictions on facilities and private gathering have been |
| $P_{11}$ | 18 Dec. 2021– 17 Jan. 2022 | Enhanced to Level 4 and restricted gatherings events to no more than 4 people |
| $P_{12}$ | 18 Jan. – 04 Mar. | Restricted gatherings events to no more than 6 people |
| $P_{13}$ | 05 Mar. – 20 Mar. | Maintaining restricted gatherings 6 people, Expanding business hours from 22:00 to 23:00 |
| $P_{14}$ | 21 Mar. – 17 Apr. | Restricted gatherings events to no more than 8 people |
| $P_{15}$ | 18 Apr. – 04 Jun. | Lifting of social distancing except mask mandates |
| $P_{16}$ | 05 Jun. – 13 Jul. | Rapidly spreading BA.4 and BA.5 subvariant as an Omicron |
| $P_{17}$ | 14 Jul. – 13 Aug. | Rapidly spreading Centaurus (BA.2.75) subvariant as an Omicron |
| $P_{18}$ | 14 Aug. – 24 Sep. | Implementation of special transportation during the summer vacation to provide a safe transportation from COVID-19 |
| $P_{19}$ | 25 Sep. – 30 Nov. | Announcement of over 90% of the population in Korea having acquired COVID-19 antibodies. |

**Supplementary Table 7.** Description of parameters in the age-structured model

| Parameter | Description | Value | Ref |
|---|---|---|---|
| $b_i$ | Time-dependent transmission rate in age group $i$ per contact | Estimation | - |
| $m_{ij}$ | Number of contacts of individual in age group $j$ with individuals in age group $i$ | Estimation | Supplementary Information C |
| $f_i$ | Case-fatality rate in age group $i$ | Estimation | - |
| $f_v$ | Case-fatality rate in age group $i$ among vaccinated people | 0.73 | [8] |
| $\phi_i$ | Vaccination allocated rate for age group $i$ | Estimation | - |
| $\sigma_S$ | Vaccine efficacy | 0.25 | [9] |
| $\sigma_I$ | Relative infectiousness of vaccinated infectious | 0.5 | [9] |
| $1/\alpha$ | Latent period (day) | 3.5 | [10] |
| $1/q$ | Period between infectiousness and hospitalization (day) | 6.8 | [10] |
| $\rho$ | Waning immunity rate ($day^{-1}$) | 1/365 | [11,12] |
| $\gamma$ | Recovered/removed rate ($day^{-1}$) | 1/10 | [13] |
| $\gamma_v$ | Vaccine-Recovered/removed rate ($day^{-1}$) | 1/7 | [14-15] |

**Supplementary Table 8.** Coefficients of contact matrixes for established periods

| Period | Time period | SD | $c_H$ | $c_W$ | $c_S$ | $c_O$ |
|---|---|---|---|---|---|---|
| $P_1$ | 26 Feb. – 11Apr. 2021 | 3 | 1.0 | 0.8 | 0.73 | 0.4 |
| $P_2$ | 12 Apr. – 17 Jun. | 3 | 1.0 | 0.8 | 0.73 | 0.4 |
| $P_3$ | 18 Jun. – 30 Jun. | 3 | 1.0 | 0.8 | 0.79 | 0.4 |
| $P_4$ | 01 Jul. – 11 Jul. | 2 | 1.0 | 0.9 | 0.80 | 0.8 |
| $P_5$ | 12 Jul. – 05 Sep. | 4 | 1.0 | 0.7 | 0.32 | 0.4 |
| $P_6$ | 06 Sep. – 17 Sep. | 4 | 1.0 | 0.7 | 0.66 | 0.6 |
| $P_7$ | 18 Sep. – 22 Sep. | 4 | 1.0 | 0.7 | 0.00 | 2.0 |
| $P_8$ | 23 Sep. – 17 Oct. | 4 | 1.0 | 0.7 | 0.81 | 0.6 |
| $P_9$ | 18 Oct. – 31 Oct. | 4 | 1.0 | 0.7 | 0.83 | 0.8 |
| $P_{10}$ | 01 Nov. – 17 Dec. | 1 | 1.0 | 1.0 | 0.85 | 1.0 |
| $P_{11}$ | 18 Dec.– 17 Jan. 2022 | 4 | 1.0 | 1.0 | 0.85 | 0.4 |
| $P_{12}$ | 18 Jan. – 04 Mar. | 4 | 1.0 | 1.0 | 0.85 | 0.6 |
| $P_{13}$ | 05 Mar. – 20 Mar. | 4 | 1.0 | 1.0 | 0.85 | 0.6 |
| $P_{14}$ | 21 Mar. – 17 Apr. | 4 | 1.0 | 1.0 | 0.85 | 0.8 |
| $P_{15}$ | 18 Apr. – 04 Jun. | - | 1.0 | 1.0 | 0.85 | 1.0 |
| $P_{16}$ | 05 Jun. – 13 Jul. | - | 1.0 | 1.0 | 0.85 | 1.0 |
| $P_{17}$ | 14 Jul. – 13 Aug. | - | 1.0 | 1.0 | 0.85 | 1.0 |
| $P_{18}$ | 14 Aug. – 24 Sep. | - | 1.0 | 1.0 | 0.85 | 1.0 |
| $P_{19}$ | 25 Sep. – 30 Nov. | - | 1.0 | 1.0 | 0.85 | 1.0 |

$c_W$ Was assumed as 1.0, 0.9, 0.8, and 0.7, corresponding to SD levels 4, 3, 2, and 1, respectively. $c_S$ indicates an average rate of school attendance [6]. $c_O =$ was determined based on 0.4, 0.6, 0.8, and 1 corresponding to the number of individuals in restricted gatherings of <5, 6, 8, and 10, respectively. Holiday ($P_7$) was assumed as 2 owing to the enhanced intervention implemented in Korea. '-' signifies the complete lifting of social distancing.

**Supplementary Table 9.** Initial conditions attained by fitting the age-structured model from July 16, 2020 to February 25, 2021

| State | 0-19 | 20-34 | 35-49 | 50-64 | 65+ |
|---|---|---|---|---|---|
| $S_i$ | 8,692,069 | 9,904,222 | 11,961,609 | 12,627,087 | 8,568,031 |
| $E_i$ | 126 | 250 | 172 | 211 | 27 |
| $I_i$ | 305 | 548 | 490 | 615 | 281 |
| $H_i$ | 708 | 1,095 | 1,205 | 1,526 | 965 |
| $R_i$ | 7,138 | 11,138 | 12,945 | 18,452 | 11,558 |
| $D_i$ | 0 | 3 | 12 | 102 | 1,255 |
| $N_i$ | 8,700,345 | 9,917,256 | 11,976,342 | 12,647,992 | 8,582,117 |

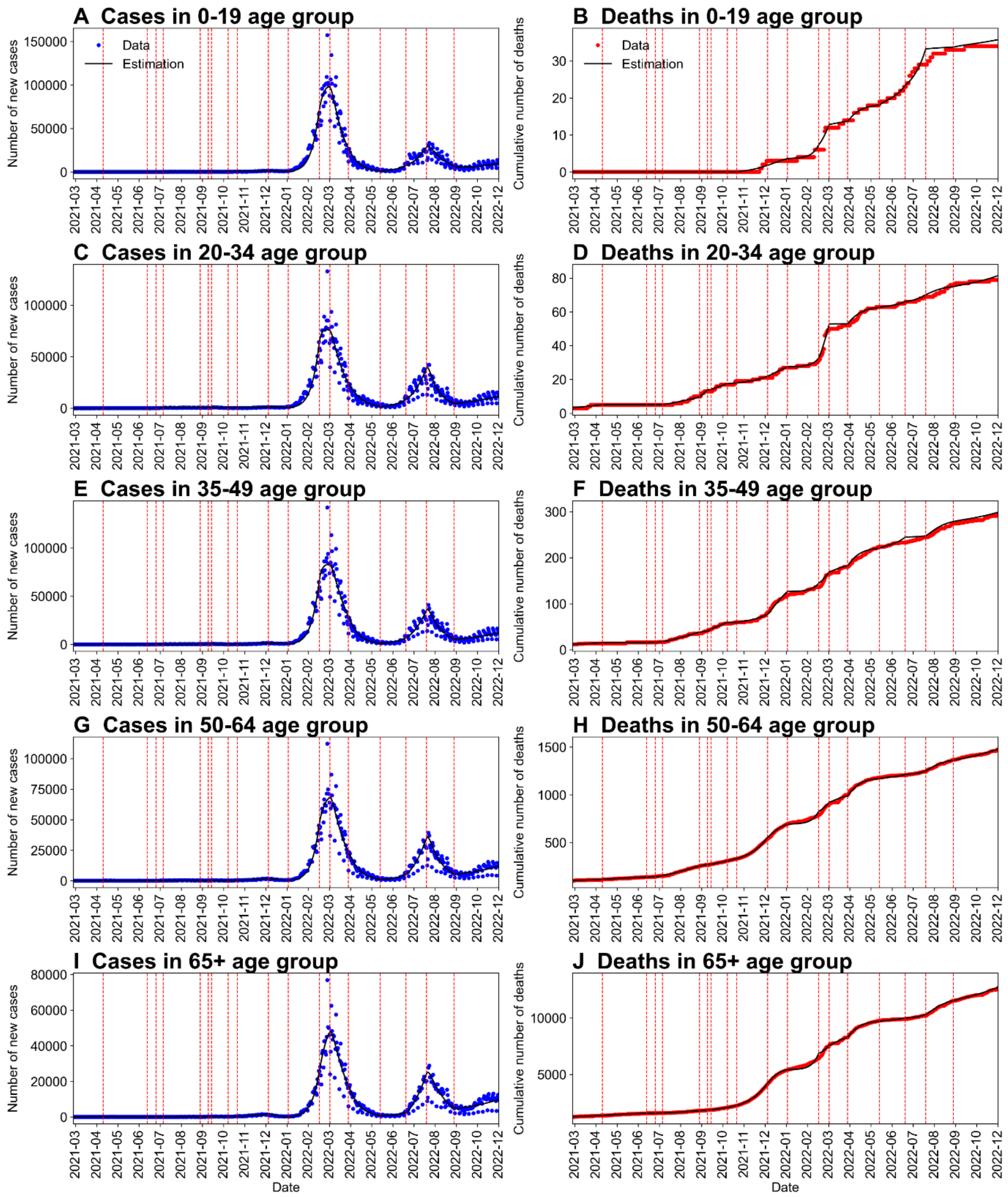


**Supplementary Figure 1. Comparison between observed data and estimated values.** The black-solid lines represent estimated values obtained using the developed model; the vertical red-dashed lines denote the periods ($P_i,\ i = 1,\cdots,19$). From the data, the blue dots in **A**, **C**, **E**, **G**, and **I** sequentially denote the daily reported cases for the five age groups. The red dots in **B**, **D**, **F**, **H**, and **J** sequentially denote the cumulative deaths for the five age groups.

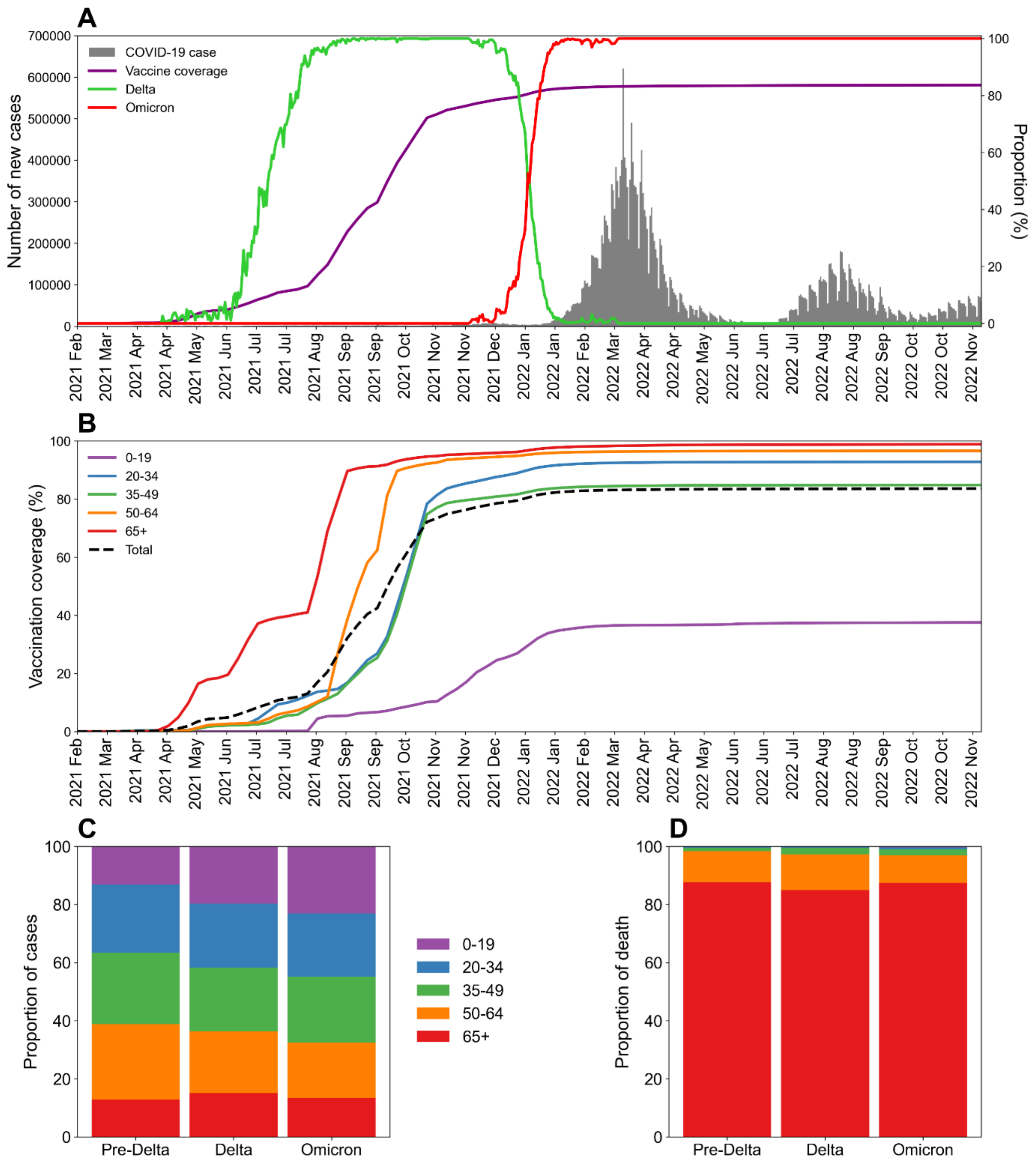


**Supplementary Figure 2.** Summary of the COVID-19 epidemiological data of the Republic of Korea from February 26 to November 30, 2022. **A:** The gray bar expresses the number of COVID-19 cases. The purple, green, and red solid lines show the proportions of vaccine coverage, Delta, and Omicron variants, respectively. **B:** The solid lines describe vaccine coverage for the five age groups, 0–19, 20–34, 35–49, 50–64, and 65+. The black dashed line represents vaccine coverage for all age groups. **C–D:** The proportions of COVID-19-related cases and deaths by age group for time phases pre-Delta, Delta, Omicron).

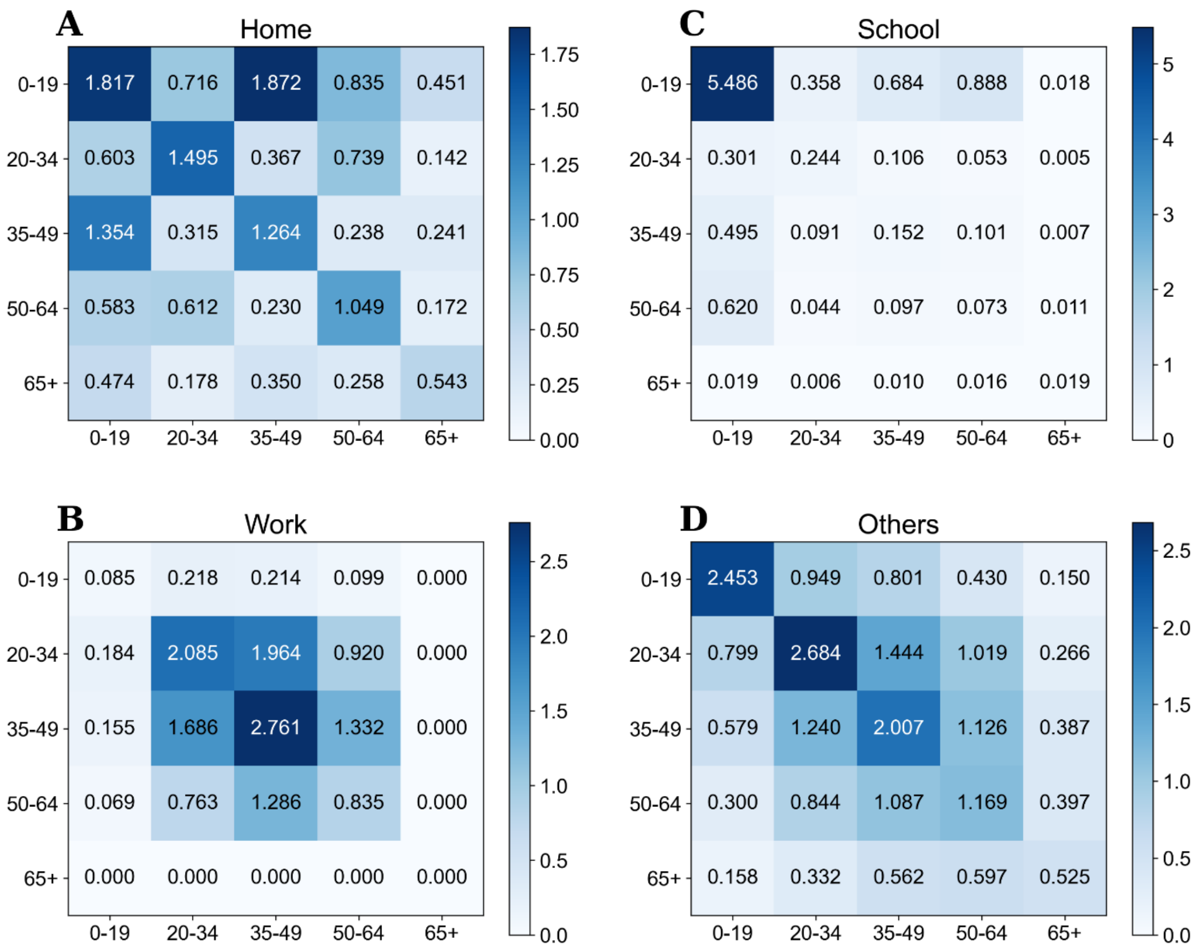


**Supplementary Figure 3.** Contact matrixes for each sub-contact matrix group.

# Supplementary References

[1] Bousali, M. et al. SARS-CoV-2 Molecular Transmission Clusters and Containment Measures in Ten European Regions during the First Pandemic Wave. *Life*, **11**(3):219, 2021.

[2] Monod, M. et al. Age groups that sustain resurging COVID-19 epidemics in the United States. *Science*, **371**(6536):eabe8372, 2021.

[3] Shin, M. S., Sim, B., Jang, W. M. & Lee, J. Y. Estimation of excess all-cause mortality during COVID-19 pandemic in Korea. *Journal of Korean medical science*, **36**(39), 2021.

[4] Prem, K., Cook, A. R. & Jit, M. Projecting social contact matrices in 152 countries using contact surveys and demographic data. *PLoS computational biology*, **13**(9):e1005697, 2017.

[5] McCarthy, Z. et al. Quantifying the shift in social contact patterns in response to non-pharmaceutical interventions. *Journal of Mathematics in Industry*, **10**(1):1-25, 2020.

[6] Ministry of Education, https://english.moe.go.kr/boardCnts/listRenewal.do?boardID=282&m=0502&s=english. Accessed: 2023-09-05.

[7] Public Data Portal, Republic of Korea; Available from: https://www.data.go.kr/data/15106451/fileData.do. Accessed: 2023-09-05.

[8] Tenforde, M. W. et al. Association between mRNA vaccination and COVID-19 hospitalization and disease severity. *Jama*, **326**(20):2043-2054, 2021.

[9] Tartof, S. Y. et al. Effectiveness of mRNA BNT162b2 COVID-19 vaccine up to 6 months in a large integrated health system in the USA: a retrospective cohort study. *The Lancet*, **398**(10309):1407-1416, 2021.

[10] Min, K. D. & Tak, S. Dynamics of the COVID-19 epidemic in the post-vaccination period in korea: a rapid assessment. *Epidemiology and health*, **43**, 2021.

[11] Hong, H. et al. Modeling Incorporating the Severity-Reducing Long-term Immunity: Higher Viral Transmission Paradoxically Reduces Severe COVID-19 During Endemic Transition. *Immune Network*, **22**(3), 2022.

[12] Aruffo, E. et al. Mathematical modelling of vaccination rollout and NPIs lifting on COVID-19 transmission with VOC: a case study in Toronto, Canada. *BMC Public Health*, **22**(1): 1-12, 2022.

[13] Ko, Y. et al. Multi-faceted analysis of COVID-19 epidemic in Korea considering omicron variant: mathematical modeling-based study. *Journal of Korean medical science*, **37**(26), 2022.

[14] Chatterjee, S., Sarkar, A., Karmakar, M., Chatterjee, S. & Paul, R. SEIRD model to study the asymptomatic growth during COVID-19 pandemic in India. *Indian Journal of Physics*, **95**:2575-2587, 2020.

[15] Ki, M. Epidemiologic characteristics of early cases with 2019 novel coronavirus (2019-nCoV) disease in Korea. *Epidemiology and health*, **42**, 2020.
